# Cryptic Speciation in Brazilian *Epiperipatus* (Onychophora: Peripatidae) Reveals an Underestimated Diversity among the Peripatid Velvet Worms

**DOI:** 10.1371/journal.pone.0019973

**Published:** 2011-06-10

**Authors:** Ivo S. Oliveira, Gustavo A. Lacorte, Cleusa G. Fonseca, Alfredo H. Wieloch, Georg Mayer

**Affiliations:** 1 Institute of Biology: Animal Evolution & Development, University of Leipzig, Leipzig, Germany; 2 Departmento de Biologia Geral, Instituto de Ciências Biológicas, Universidade Federal de Minas Gerais, Belo Horizonte, Minas Gerais, Brazil; 3 Departamento de Zoologia, Universidade Federal de Minas Gerais, Instituto de Ciências Biológicas, Belo Horizonte, Minas Gerais, Brazil; University of Arkanas, United States of America

## Abstract

**Background:**

Taxonomical studies of the neotropical Peripatidae (Onychophora, velvet worms) have proven difficult, due to intraspecific variation and uniformity of morphological characters across this onychophoran subgroup. We therefore used molecular approaches, in addition to morphological methods, to explore the diversity of *Epiperipatus* from the Minas Gerais State of Brazil.

**Methodology/Principal Findings:**

Our analyses revealed three new species. While *Epiperipatus diadenoproctus* **sp. nov.** can be distinguished from *E. adenocryptus* **sp. nov.** and *E. paurognostus* **sp. nov.** based on morphology and specific nucleotide positions in the mitochondrial *cytochrome c oxidase subunit I* (*COI*) and *small ribosomal subunit RNA* gene sequences (*12S rRNA*), anatomical differences between the two latter species are not evident. However, our phylogenetic analyses of molecular data suggest that they are cryptic species, with high Bayesian posterior probabilities and bootstrap and Bremer support values for each species clade. The sister group relationship of *E. adenocryptus* **sp. nov.** and *E. paurognostus* **sp. nov.** in our analyses correlates with the remarkable morphological similarity of these two species. To assess the species status of the new species, we performed a statistical parsimony network analysis based on 582 base pairs of the *COI* gene in our specimens, with the connection probability set to 95%. Our findings revealed no connections between groups of haplotypes, which have been recognized as allopatric lineages in our phylogenetic analyses, thus supporting our suggestion that they are separate species.

**Conclusions/Significance:**

Our findings suggest high cryptic species diversity and endemism among the neotropical Peripatidae and demonstrate that the combination of morphological and molecular approaches is helpful for clarifying the taxonomy and species diversity of this apparently large and diverse onychophoran group.

## Introduction

The phylogeny and taxonomy of the neotropical Peripatidae is understudied [1]–[4] and the estimated number of 70–80 described species and subspecies apparently does not reflect the actual diversity of the group [5]. The major difficulties with the peripatid taxonomy arise from intraspecific variation and uniformity of morphological characters – an issue that could be addressed by applying molecular techniques, in addition to classical morphological methods. While scanning electron microscopy has revealed a high morphological diversity of the neotropical Peripatidae (e.g., [1], [2], [4]), molecular methods have not been used to clarify the genetic diversity of the group. However, these methods have shown that cryptic speciation is a common phenomenon in the Peripatopsidae, another large onychophoran taxon [6]–[14].

To provide a basis for future research on the neotropical Peripatidae, we applied molecular and morphological methods, including scanning electron microscopy, and analysed specimens from four different localities of the Minas Gerais State of Brazil. Our data suggest cryptic speciation and high endemism in the neotropical Peripatidae and provide evidence of three new species of *Epiperipatus*, for which we provide formal descriptions and type designations to fulfil the requirements of the International Code of Zoological Nomenclature (ICZN).

## Results

### General anatomy of the specimens studied

**Figure 1:**
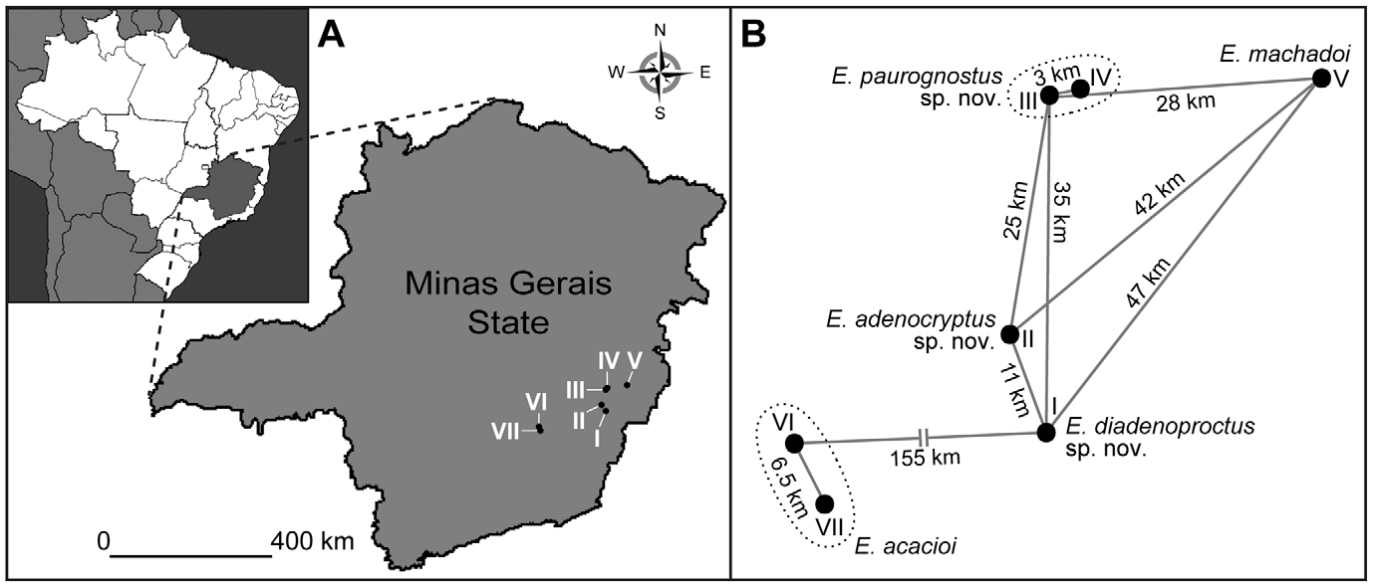
Distribution of onychophoran species in the Minas Gerais State of Brazil, including the three new species described herein. A, Overview. Species localities numbered as follows: I, Reserva Particular do Patrimônio Natural (RPPN) Mata do Sossego (type locality of *Epiperipatus diadenoproctus* sp. nov.); II, Córrego dos Ferreiras (type locality of *E. adenocryptus* sp. nov.); III, Mata do Eremitério (type locality of *E. paurognostus* sp. nov.); IV, Rancho Primavera (additional locality of *E. paurognostus* sp. nov.); V, RPPN Feliciano Miguel Abdala (type locality of *E. machadoi*); VI, Estação Ecológica de Tripuí (type locality of *E. acacioi*); VII, Parque Estadual do Itacolomi (additional locality of *E. acacioi*). B, Diagram illustrating air-line distances between the localities of each species occurring in the Minas Gerais State.

**Figure 2:**
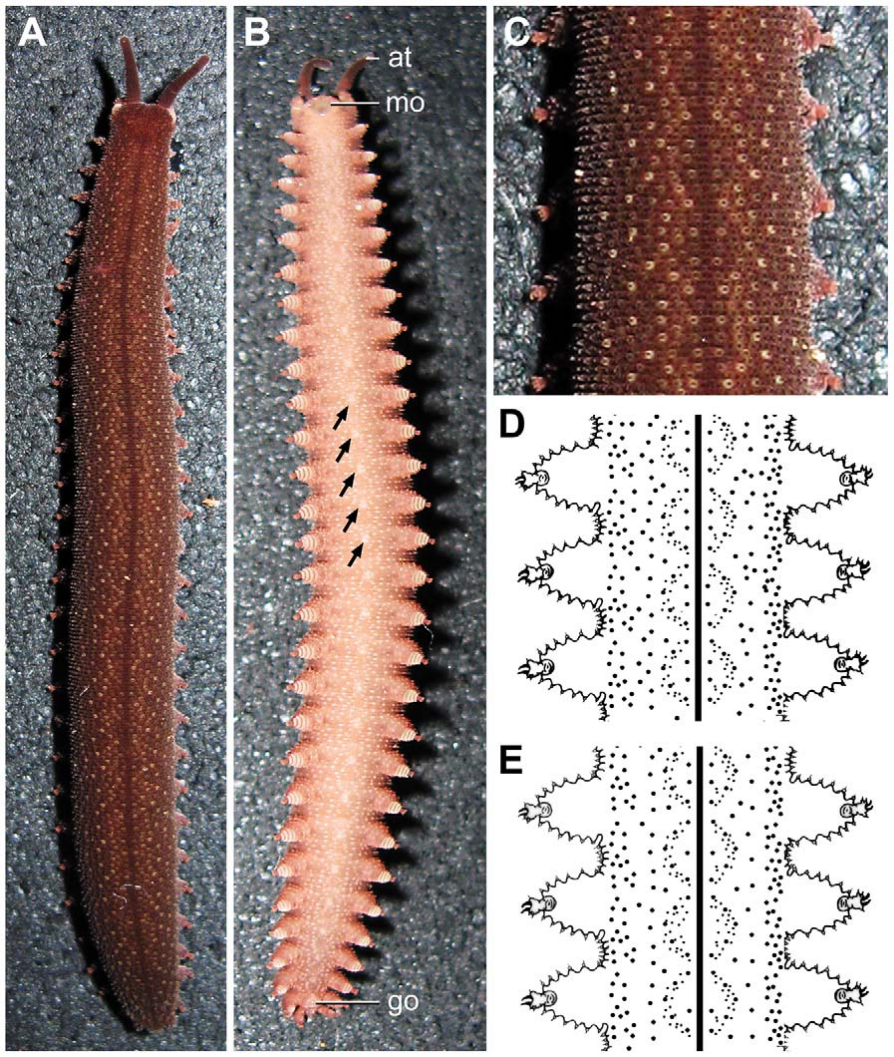
Body colour pattern in onychophoran specimens studied, exemplified by *Epiperipatus diadenoproctus* sp. nov. Photographs (A–C) and ink drawings (D, E). A, Dorsal colour pattern in an anaesthetised specimen. B, Ventral colour pattern in the same specimen. Arrows point to the ventral organs. C, Detail of dorsal colour pattern. D, E, Diagrams showing slight variation of dorsal body colour pattern. D, Pattern with two pairs of large papillae in each circle. E, Pattern with either missing or a variable number of large papillae in each circle. Abbreviations: at, antenna; go, genital opening; mo, mouth.

Since there are gaps in our knowledge of morphological characters in representatives of *Epiperipatus* (Table S1), we examined and compared in detail *Epiperipatus* specimens from four different localities in the Minas Gerais State of Brazil (numbered I to IV in Figure 1A, B). The ground colour of all specimens is *in vivo* brown, with numerous light-brown papillae spread over the body surface (Figure 2A, C). In addition, there is a repeated pattern of bilateral light-brown arcs on each side of the dark-brown dorsal midline, which consist of five to six large and numerous small light-brown dermal papillae (Figure 2C–E; Figure S1A, C). The arcs form repeated circles enclosing one or two pairs of additional large, light-brown primary papillae that are, however, missing in some circles (Figure 2C–E). The dorsal body surface of fixed specimens is greyish-brown and shows the same pattern as in living specimens. The ventral body surface is *in vivo* pinkish-beige. The ventral organs are brighter and clearly visible (Figure 2B; Figure S1B, D).

**Figure 3:**
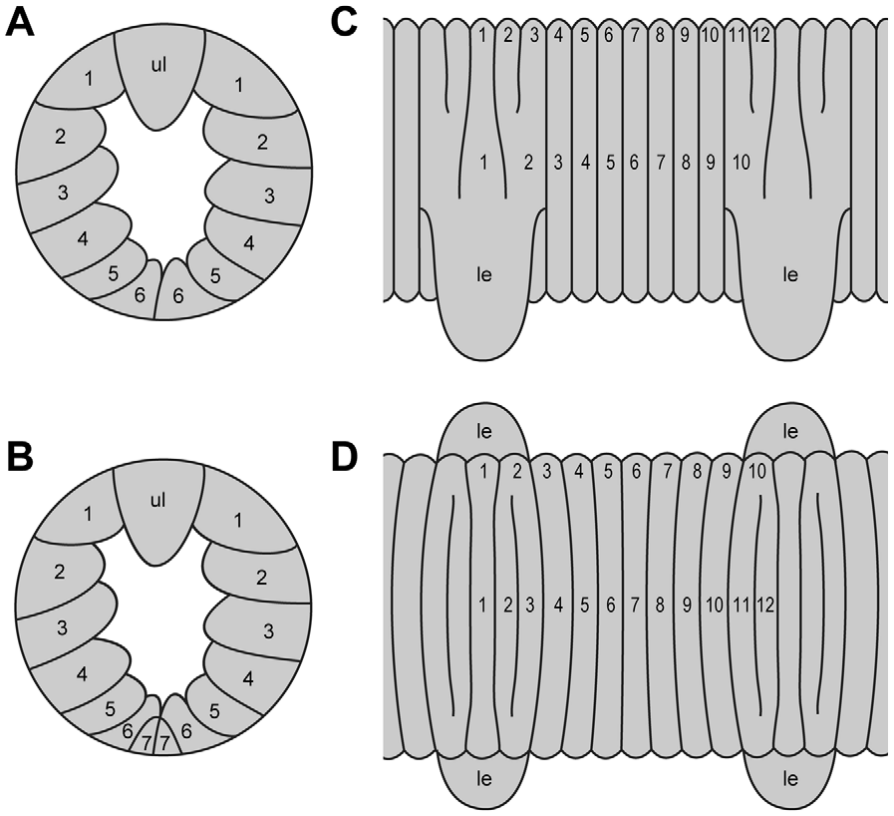
Diagrammatic arrangement of oral lips and plicae in onychophoran specimens studied. A, Single unpaired and six paired lips (numbered). Anterior is up. B, Single unpaired and seven paired lips (numbered). Anterior is up. C, Arrangement of plicae (numbered) in lateral view. D, Arrangement of plicae (numbered) in dorsal view. Note two pairs of anastomosing plicae in each leg-bearing segment. Abbreviations: le, legs; ul, unpaired anterior lip.

**Figure 4:**
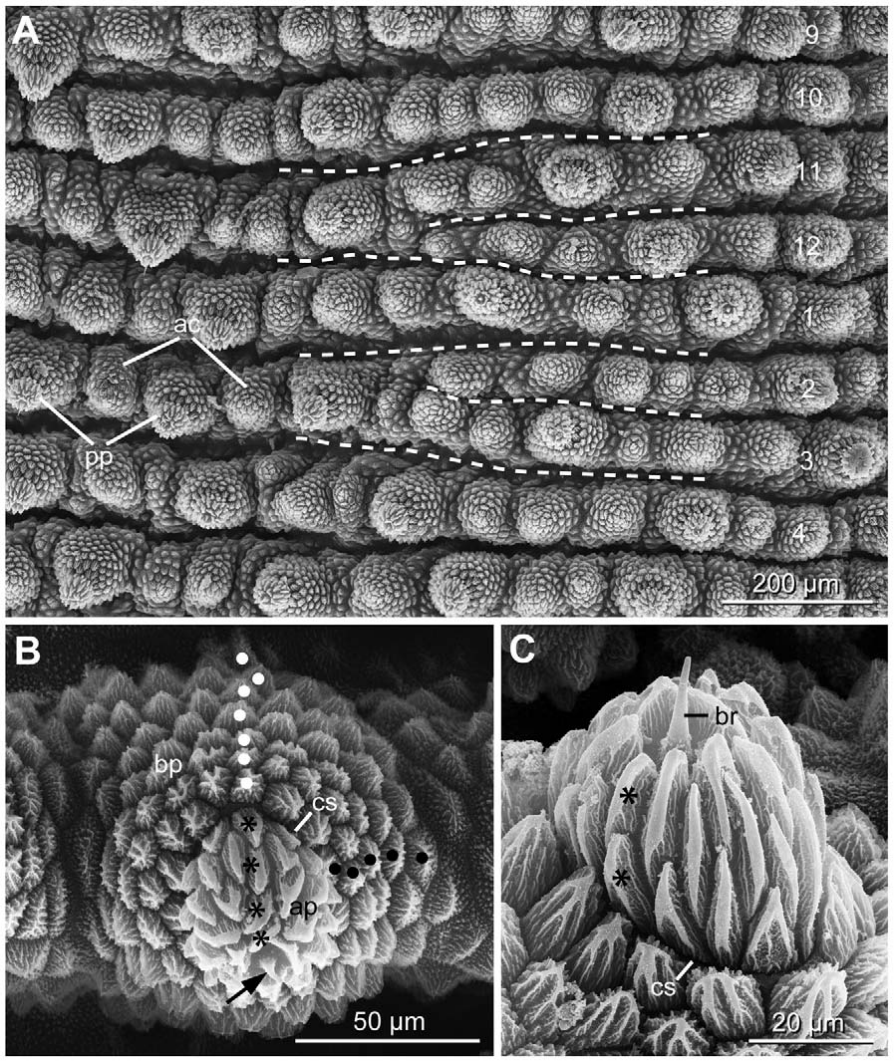
Arrangement of plicae and structure of dermal papillae in onychophoran specimens studied. Scanning electron micrographs. A, Portion of dorsolateral integument in *E. adenocryptus* **sp. nov.** showing plical anastomoses. The plicae are numbered. Anterior is up, median is right. B, Dorsal primary papilla in *E. adenocryptus* **sp. nov.** showing five lateral (black dots) and seven anterior scale ranks (white dots) in the basal piece and four anterior scale ranks in the apical piece (asterisks). Anterior is up, median is left. Note the posteriorly displaced sensory bristle (arrow). C, Detail of an asymmetrical apical piece in *E. paurognostus* **sp. nov.** showing only two posterior scale ranks (asterisks) and a thorn-shaped and posteriorly displaced bristle. Abbreviations: ac, accessory papillae; ap, apical piece; bp, basal piece; br, sensory bristle; cs, constriction between apical and basal pieces; pp, primary papillae.

The antennal tip consists of 13 rings (including the terminal button), with the 9^th^, 11^th^ and 13^th^ rings thinner than the others. The eyes and the frontal organs are well-developed. The mouth is surrounded by six to seven pairs of oral lips and one unpaired, large anterior lip (Figure 3A, B). The dorsomedian furrow is distinct along the entire body and the hyaline organs are present. The dorsal integument shows 12 plicae per segment, four of which (2^nd^ with 3^rd^ and 11^th^ with 12^th^) anostomose with each other towards each side of the body (Figures 3C, D and 4A). Thus, only 10 plicae per segment are seen laterally and only seven of them (3^rd^ to 9^th^) pass to the ventral side between each two subsequent leg pairs (Figure 3C, D).

There are one to two accessory papillae between each two primary papillae in the dorsal integument (Figure 4A), but a variable number of two to six adjacent accessory papillae are found along the dorsal midline (Figure S2A–F). The primary papillae show roundish bases and vary in size. A distinct constriction separates the apical and basal pieces (Figure 4B, C). The basal pieces possess five to six lateral and seven to eight anterior scale ranks (Figure 4B). The apical pieces are asymmetrical, with three to four anterior and two to three posterior scale ranks (Figure 4B, C). Sensory bristles are thorn-shaped and displaced posteriorly (Figure 4C). The primary papillae at the level of legs and their apical pieces are elongated and possess slender scales.

**Figure 5:**
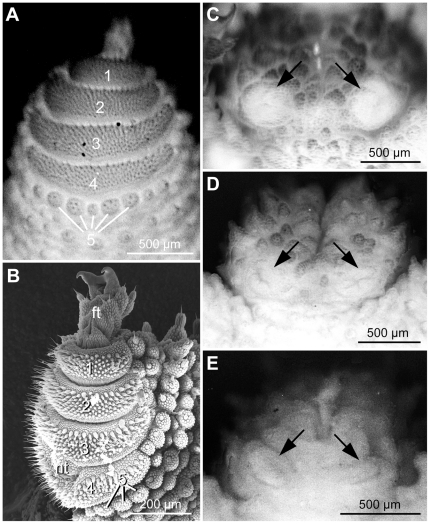
Structure of spinous pads and male anal gland papillae in onychophoran specimens studied. Light micrographs (A, C–E) and scanning electron micrograph (B). A, Leg from the mid-body of *E. diadenoproctus* **sp. nov.** in ventral view. Spinous pads are numbered. Note the presence of a fifth fragmented pad. B, Distal portion of fifth leg of *E. paurognostus* **sp. nov.** showing nephridial tubercle, complete fourth pad and fragmented fifth pad. C–E, Posterior ends in males of *E. diadenoproctus* **sp. nov.** (C), *E. adenocryptus* **sp. nov.** (D) and *E. paurognostus* **sp. nov.** (E) showing anal gland papillae (arrows). Note the well-developed, roundish anal gland papillae in *E. diadenoproctus* **sp. nov.** Abbreviation: ft, foot; nt, nephridial tubercle.

Each leg shows eight plical rings and four complete spinous pads and a fifth fragmented pad (Figure 5A), but two posterior leg pairs are reduced in size and bear only three complete spinous pads and a fourth fragmented pad. Most feet have two anterior and one posterior foot papillae, but some of them show only one anterior and one posterior or only two anterior papillae. Each proximal and distal setiform ridges on the ventral surface of the foot possess one or two bristles. Eversible coxal vesicles are present at the bases of most legs, except for the fourth and fifth leg pairs, which show nephridial tubercles in a distal position between the third and fourth spinous pads. The fourth pad is arched and complete (not divided by the nephridial tubercle) in these leg pairs (Figure 5B). Single crural tubercles are present in two pre-genital leg pairs in males. The genital opening lies mid-ventrally in the segment of the penultimate leg pair. The male genital pad is divided by a single longitudinal furrow in two compartments whereas the female genital pad is divided by two perpendicular furrows in four compartments.

### Morphological differences between the specimens studied

Despite our detailed morphological analysis, including scanning electron microscopy, we found only a few morphological characters that differ between the specimens from different localities. In particular, the anatomy of the anal gland papillae differs in males from the Particular Reserve of Natural Patrimony ( = RPPN) Mata do Sossego (*E. diadenoproctus* **sp. nov.**: locality I in Figure 1A, B) compared to those found at the three other localities (*E. adenocryptus* **sp. nov.**: locality II, and *E. paurognostus* **sp. nov.**: localities III and IV in Figure 1A, B). The anal gland papillae in specimens of *E. diadenoproctus* **sp. nov.** are large, roundish and brighter than the surrounding integument (Figure 5C) whereas they are smaller, bean-shaped and hardly visible in males from other localities (Figure 5D, E). Furthermore, the specimens of *E. diadenoproctus* **sp. nov.** are characterised by different numbers of leg pairs, with some males showing 28 leg pairs whereas specimens of *E. adenocryptus* **sp. nov.** and *E. paurognostus* **sp. nov.** with the same number of leg pairs are all females (Table 1). Thus, while *E. diadenoproctus* **sp. nov.** can be distinguished morphologically, we did not find any unambiguous distinctive characters between *E. adenocryptus* **sp. nov.** and *E. paurognostus* **sp. nov.** Although the numbers of leg pairs in each sex differ between the two species, with overlapping numbers in *E. paurognostus* **sp. nov.** (Table 1), this result has to be corroborated by using a large number of specimens. Nevertheless, *E. adenocryptus* **sp. nov.** and *E. paurognostus* **sp. nov.** can be distinguished unambiguously by applying molecular methods.

**Table 1:** Numbers of leg pairs in specimens of each sex in the three new onychophoran species.

Species	Sex	Number of leg pairs				
		26	27	28	29	30
*E. diadenoproctus* **sp. nov.**	♂♂	4	20*	6	-	-
	♀♀	-	-	-	28	3
*E. adenocryptus* **sp. nov.**	♂♂	2	15	-	-	-
	♀♀	-	-	3	12	2
*E. paurognostus* **sp. nov.**	♂♂	5	11	-	-	-
	♀♀	-	1	4	8	-

*Including one male, which shows an asymmetrical number of 27 and 28 legs on each side of the body.

### Analyses of molecular data

The amplified *cytochrome c oxidase subunit I* (*COI*) fragments in all specimens studied were 590 bp long. However, the sequence ends of some fragments were of suboptimal quality and, therefore, had to be excluded from our analysis so that the final alignment contained 582 bp. Among 159 variable sites, 61 were parsimoniously informative (Figure S3). The average base frequencies were A + T biased, in particular in the third codon position. Of all variable sites, 68% were substituted in the third codon position, while 20% showed substitutions in the first and 12% in the second position. The translation of *COI* nucleotide sequences into amino acid sequences revealed no stop codons, suggesting that all sequences belong to functional mitochondrial protein-coding genes. Furthermore, the alignment of the amino acid sequences shows that of 194 amino acids, only 42 are variable (Figure S4). The amplified *small ribosomal subunit RNA* (*12S rRNA*) fragments were 355 bp long. Their alignment revealed 15 gap sites distributed throughout the fragment lengths, which had to be excluded from our analysis to avoid the necessity of entering a new character state. Among 93 variable sites, 61 were parsimoniously informative (Figure S5). Like in the *COI* sequences, an A + T bias was found also in the *12S rRNA* sequences (Table S2).

According to our data, the genetic distances range from 4.4% to 9.6% (including the Brazilian species only) and from 4.4% to 18.6% (including all species studied). *Epiperipatus adenocryptus* **sp. nov.** displays the highest mean intraspecific genetic distance (2.0%), while *E. paurognostus* **sp. nov.** shows the lowest value (1.0%) (Table S3). *Epiperipatus paurognostus* **sp. nov.** and *E. diadenoproctus* **sp. nov.** are the most divergent species, with high interspecific distance values in all pair-wise comparisons, while *E. adenocryptus* **sp. nov.** and *E. paurognostus* **sp. nov.** show the lowest interspecific genetic distances (Table S3).

**Figure 6:**
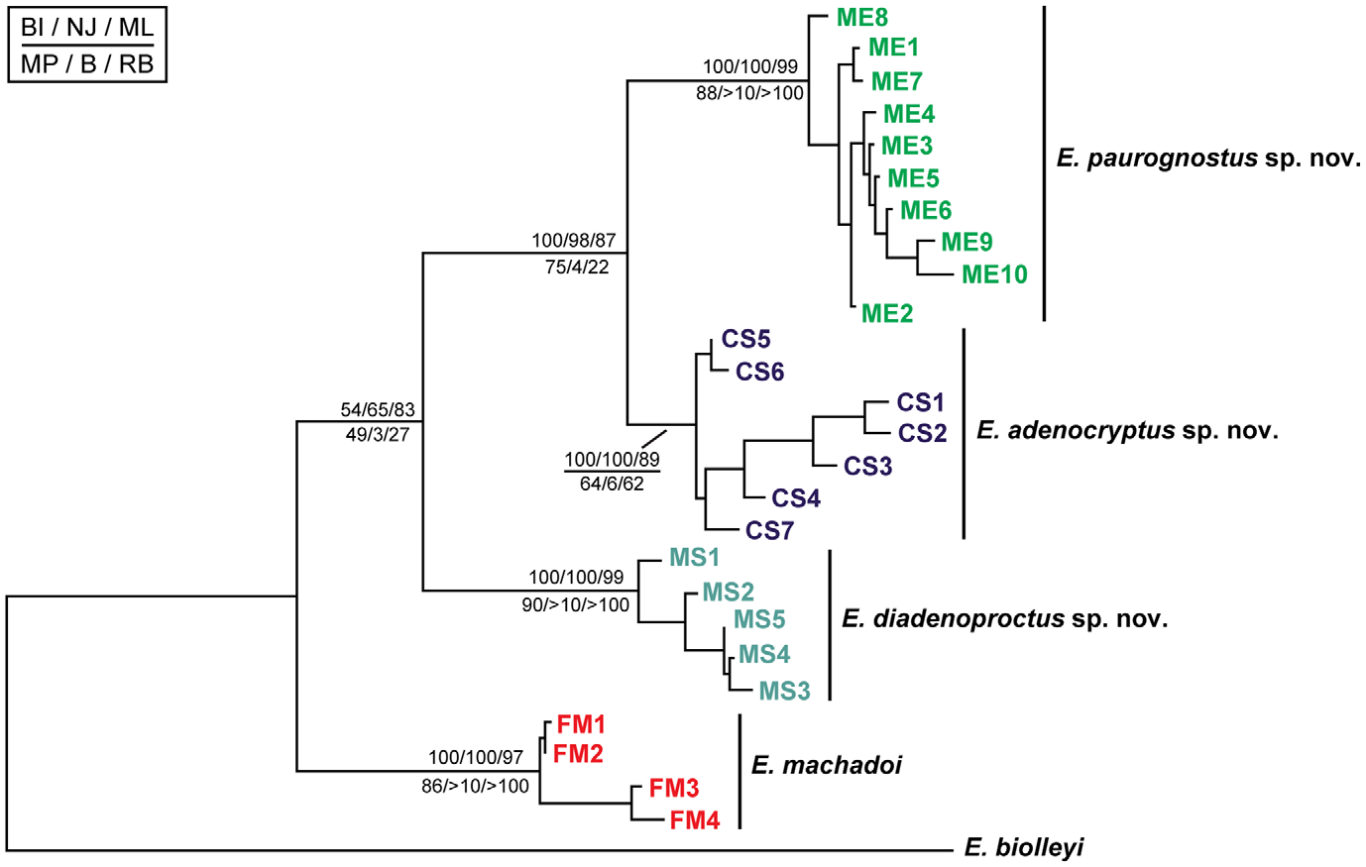
Maximum Likelihood topology amongst onychophoran specimens from different localities. Combined mitochondrial data sets (*COI + 12S rRNA*), with *E. biolleyi* as an outgroup. Bayesian posterior probabilities and bootstrap values are given in the following order: BI/NJ/ML//MP/Bremer/Relative Bremer index decay. Abbreviations: CS, Córrego dos Ferreiras; FM, RPPN Feliciano Miguel Abdala; ME, Mata do Eremitério; MS, RPPN Mata do Sossego.

We used four different methods, Neighbor-Joining (NJ), Maximum Parsimony (MP), Maximum Likelihood (ML) and Bayesian Inference (BI), for phylogenetic analyses, which all revealed similar topologies and the same monophyletic clades for the hypothesised species (Figure 6; Figures S6, S7, S8, and S9). The monophyly of each species is well-supported, except for a low bootstrap support value (64%) for *E. adenocryptus* **sp. nov.** in the MP topology (Figure S6). In all analyses, *E. adenocryptus* **sp. nov.** sister groups with *E. paurognostus* **sp. nov.** The node supporting the clade uniting *E. diadenoproctus* **sp. nov.**, *E. adenocryptus* **sp. nov.** and *E. paurognostus* **sp. nov.** shows low Bayesian posterior probabilities (54) (Figure 6; Figure S7) and bootstrap support values in both MP and NJ topologies (49% and 65%, respectively) (Figure 6; Figures S6 and S8).

**Figure 7:**
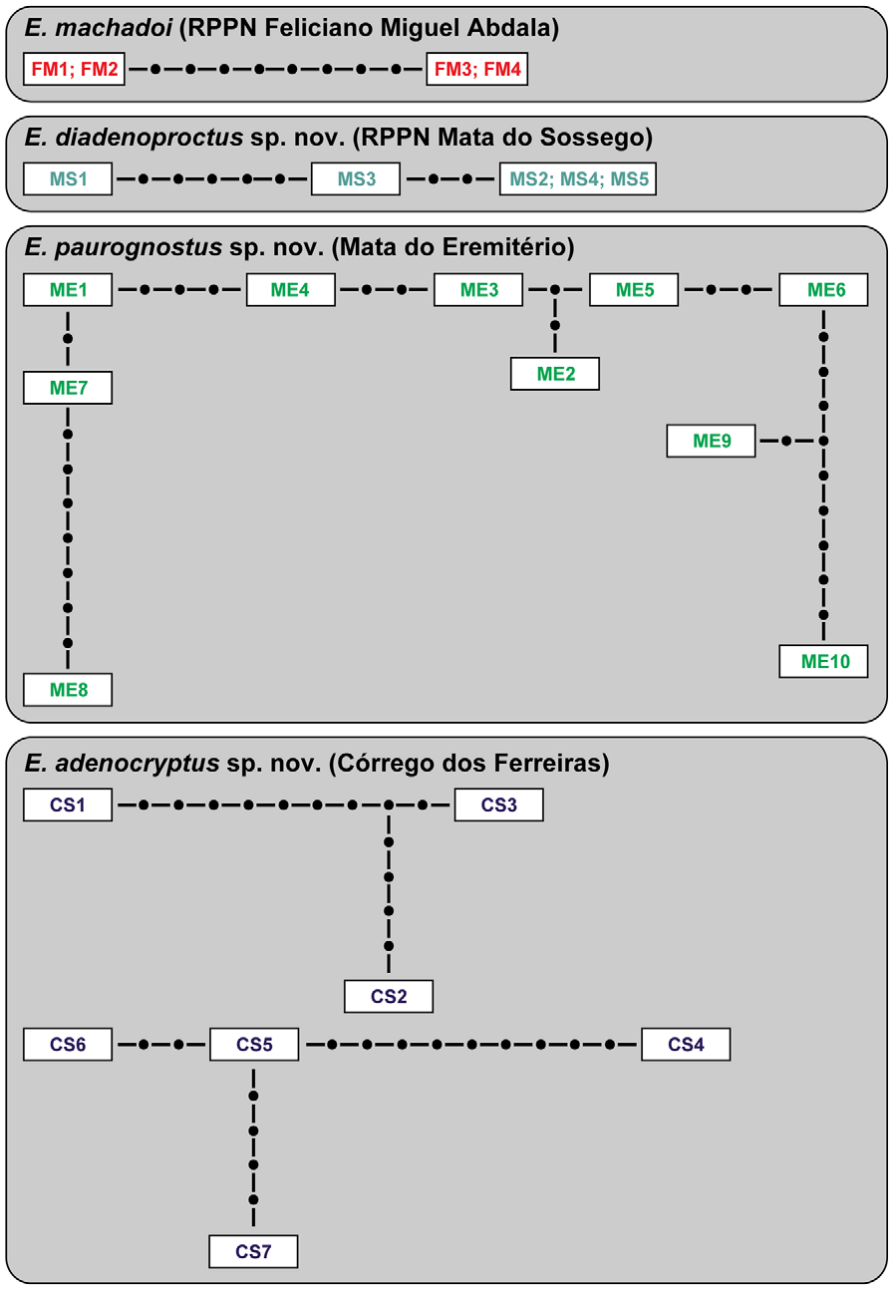
Haplotype networks for the *COI* sequences of *Epiperipatus* specimens from different localities. Abbreviations as per Table 2. The connection probability was set to 95% (see ref. [48]). Each dot indicates one missing or unsampled haplotype. Two or more names in one frame represent identical genotypes.

Our statistical parsimony network analyses revealed 22 haplotypes and five separate networks among the *COI* sequences of the 26 specimens studied (Figure 7). Each network includes specimens from a single location, except for *E. adenocryptu*s **sp. nov.**, which forms two separate haplotype networks from the same locality. These findings correspond well to the results of our phylogenetic analyses and support the existence of three new allopatric species.

**Table 2:** Origin of onychophoran specimens sequenced and corresponding GenBank accession numbers.

Taxon	Collecting cite	Specimen	GenBank accession number (*COI*)	GenBank accession number (*12S rRNA*)
*E. adenocryptus* **sp. nov.**	Córrego dos Ferreiras	CS1	HQ236108	HQ236134
		CS2	HQ236109	HQ236135
		CS3	HQ236110	HQ236136
		CS4	HQ236111	HQ236137
		CS5	HQ236112	HQ236138
		CS6	HQ236113	HQ236139
		CS7	HQ236114	HQ236140
*E. diadenoproctus* **sp. nov.**	RPPN Mata do Sossego	MS1	HQ236093	HQ236119
		MS2	HQ236094	HQ236120
		MS3	HQ236095	HQ236121
		MS4	HQ236096	HQ236122
		MS5	HQ236097	HQ236123
*E. machadoi*	RPPN Feliciano Miguel Abdala	FM1	HQ236089	HQ236115
		FM2	HQ236090	HQ236116
		FM3	HQ236091	HQ236117
		FM4	HQ236092	HQ236118
*E. paurognostus* **sp. nov.**	Mata do Eremitério	ME1	HQ236098	HQ236124
		ME2	HQ236099	HQ236125
		ME3	HQ236100	HQ236126
		ME4	HQ236101	HQ236127
		ME5	HQ236102	HQ236128
		ME6	HQ236103	HQ236129
		ME7	HQ236104	HQ236130
		ME8	HQ236105	HQ236131
		ME9	HQ236106	HQ236132
		ME10	HQ236107	HQ236133

Taken together, the results of our morphological examinations revealed only two novel morphotypes of *Epiperipatus* whereas the molecular analyses provide evidence of three new well-supported species clades, two of which are therefore cryptic (*sensu* ref. [15]). Since the three new lineages are monophyletic in all our phylogenetic analyses and form separate haplotype networks, we recognise them as separate species.

### Description of three new species of *Epiperipatus*

#### *Epiperipatus diadenoproctus* sp. nov

urn:lsid:zoobank.org:act:992197FB-7D70-4A9A-A4F3-E3D39135B89D.

*Holotype*. ♂, BRAZIL, Minas Gerais, Simonésia, RPPN Mata do Sossego, 1150 m, Atlantic rain forest, 20°04′21″S & 42°04′12″W, 15–19 July 2008, I. S. Oliveira & F. N. S. Queirós (UFMG0140).

*Paratypes*. Same data as for holotype, ♀, 7–19 March 1999, U. Caramaschi *et al.* (MNRJ0012); 1♂, 3♀♀, 28 June 2008, I. S. Oliveira & S. Genelhú (UFMG0096-99); 29♂♂, 27♀♀, 15–19 July 2008, I. S. Oliveira & F. N. S. Queirós (UFMG0100-106, 108–0139 & 0141–0157).

*Etymology.* The name *diadenoproctus* is derived from Greek δύο ( =  two), αδένες ( =  glands) and πρ*ω*κτός ( =  anus) [16], in reference to paired anal gland papillae present in males (Figure 5C).

*Diagnosis.* Anal gland papillae well-developed, roundish and brighter than surrounding integument (Figure 5C); 26–28 leg pairs in males and 29–30 in females, without overlap between sexes (Table 1). *COI* and *12S rRNA* sequences as in specimens MS1-MS5 (Table 2). The species is characterised by 14 specific nucleotide positions: four in the *COI* sequence and 10 in the *12S rRNA* sequence (Table 3). Phylogenetic relationship as in Figure 6; intra- and interspecific distances as in Table S3.

**Table 3:** Unambiguous synapomorphies (character states in parentheses) of each *Epiperipatus* species studied.

Species	Synapomorphies*				
	N	COI			12S rRNA
		1st**	2nd**	3rd**	
E. adenocryptus sp. nov.	7	457(C); 583(A)	479(T)	495(G); 666(G) 708(T)	425(G)
E. diadenoproctus sp. nov.	14			369(G); 381(A) 534(A); 555(T)	127(A); 154(T); 173(A) 237(G); 260(G); 288(A) 294(A); 296(G); 328(G) 337(G)
E. machadoi	18	238(A)		237(G); 270(T) 273(C); 363(A) 429(G); 417(T) 489(A); 495(T) 510(A); 537(A) 600(T)	204(G); 207(T); 215(A); 269(A); 278(A); 362(T)
E. paurognostus sp. nov.	10	241(T); 274(T)	242(T); 257(G) 278(T)	249(T); 258(C) 261(C); 270(C)	425(C)

N  =  total number of synapomorphies.

^*^The nucleotide positions are based on complete *COI* and *12S rRNA* sequences of *E. biolleyi* (GenBank: DQ666064).

^**^Codon position.

*Description.* Body length after fixation 10.5–44.5 mm, width 1.6–5.0 mm, height 1.2–3.5 mm. Juveniles reddish-brown and without any pattern. Antennae with 38 to 43 rings, 25 to 30 of which in antennal body and remaining 13 in antennal tip (including terminal button). Outer jaw blade with one principal tooth and one or two accessory teeth (Figure S10A); inner jaw blade with one principal tooth, two accessory teeth and 10 denticles (Figure S10B); accessory teeth with straight and parallel anterior and posterior faces and a convex ventral face, forming acute angle with posterior face and obtuse angle with anterior face. Remaining characters, shared with *E. adenocryptus* **sp. nov.** and *E. paurognostus* **sp. nov.**, as described above (see the Results section entitled “General anatomy of the specimens studied”).

*Remarks on anomalies.* One male shows an asymmetrical number of 27 and 28 legs on each body side (Table 1) and another male has doubled crural tubercles on a pre-genital leg.

*Remarks on habitat preference.* The juveniles of *E. diadenoproctus* **sp. nov.** inhabit leaf litter whereas the adults occur either under or within rotten logs. In addition, adult specimens were found in human rubbish (roof clay tiles) placed in front of a researchers' accommodation, close to the border of the investigated forest fragment. According to the locals, the roof tiles and rubbish had remained untouched for five years and contained a diverse invertebrate fauna. In contrast to the animals found in human rubbish, specimens collected in the forest were always solitary and a gregarious behaviour was not observed.

*Distribution. Epiperipatus diadenoproctus* **sp. nov.** occurs only at the type locality, the RPPN Mata do Sossego (locality I in Figure 1A, B). A re-examination of the material identified as “*Peripatus* sp. 3” reported from RPPN Mata do Sossego and from three other localities by Sampaio-Costa *et al.* [5] revealed that only the specimens from Mata do Sossego, the type locality, belong to *E. diadenoproctus* **sp. nov.**

#### *Epiperipatus adenocryptus* sp. nov

urn:lsid:zoobank.org:act:D172B531-2CFA-4128-B220-DD45AE2218BB.

*Holotype*. ♂, BRAZIL, Minas Gerais, Santa Bárbara do Leste, Córrego dos Ferreiras, 1050 m, Atlantic rain forest, 42°06′46.9758″W & 19°58′59.24619″S, 17 June 2008, I. S. Oliveira & S. Genelhú (UFMG0071).

*Paratypes*. Same data as for holotype, ♀, 01 November 2003, E. T. Silva (UFMG0016); 11♂♂, 12♀♀, 17 June 2008, I. S. Oliveira & S. Genelhú (UFMG0070; 0072-0091).

*Etymology.* The name *adenocryptus* is derived from Greek αδένες ( =  glands) and κρυπτ*ο*ς ( =  hidden) [16], in reference to hardly visible anal gland papillae in males (Figure 5D).

*Diagnosis.* Anal gland papillae poorly developed, bean-shaped, hardly visible and similar in colour to surrounding integument (Figure 5D); 26–27 leg pairs in males and 28–30 in females, without overlap between sexes (Table 1). *COI* and *12S rRNA* sequences as in specimens CS1–CS7 (Table 2). The species is characterised by six specific nucleotide positions in the *COI* sequence and a single position in the *12S rRNA* sequence (Table 3). Phylogenetic relationship as in Figure 6; intra- and interspecific distances as in Table S3.

*Description.* Body length after fixation 12.3–43.0 mm, width 1.7–4.4 mm, height 1.1–3.6 mm. Juveniles reddish-brown and with the same pattern as in adults. Antennae with 38 to 43 rings, 25 to 30 of which in antennal body and remaining 13 in antennal tip (including terminal button). Outer jaw blade with one principal and one accessory teeth (Figure S10C); inner jaw blade with one principal tooth, one or two accessory teeth and seven denticles (Figure S10D); first (anterior-most) accessory tooth larger than second and with straight and parallel anterior and posterior faces; ventral face straight or convex, forming acute angle with posterior face and obtuse angle with anterior face; second accessory tooth thorn-shaped; asymmetric number of accessory teeth (either one or two) on inner jaws of left and right body sides in some specimens. Remaining characters, shared with *E. diadenoproctus* **sp. nov.** and *E. paurognostus* **sp. nov.**, as described above (see the Results section entitled “General anatomy of the specimens studied”).

*Remarks on habitat preference.* Adults and juveniles of *Epiperipatus adenocryptus* **sp. nov.** are found in leaf litter and inside rotten logs.

*Distribution. Epiperipatus adenocryptus* **sp. nov.** has been recorded only from the type locality, Córrego dos Ferreiras (locality II in Figure 1A, B).

#### *Epiperipatus paurognostus* sp. nov

urn:lsid:zoobank.org:act:CC6B56A8-6DA8-4BB6-80FD-E2960EE356F2.

*Holotype.* ♂, BRAZIL, Minas Gerais, Piedade de Caratinga, Mata do Eremitério, 897 m, Atlantic rain forest, 42°05′22.75085″W & 19°45′33.97023″S, 14 June 2008, I. S. Oliveira & S. Genelhú (UFMG0065).

*Paratypes.* Same data as for holotype, ♀, 31 May 2008, H. Coelho & S. Genelhú (UFMG0057); 2♂♂, 7♀♀, 14 June 2008, I. S. Oliveira & S. Genelhú (UFMG0058-0064 & UFMG0066); 1♂, 2♀♀, 22 July 2009, I. S. Oliveira & G. A. Lacorte (UFMG0179–0181); 1♂, Piedade de Caratinga, Rancho Primavera, 830 m, Atlantic rain forest, 42°3′35.54″W–19°45′4.43″S, 23 April 2009, H. Coelho (UFMG0184).

*Etymology.* The name *paurognostus* is derived from Greek παυρ*ο*ς ( =  little) and γν*ω*στός ( =  distinguished) [16], in reference to a remarkable morphological similarity of *E. paurognostus* **sp. nov.** to *E. adenocryptus* **sp. nov.**, which makes it difficult to distinguish the species.

*Diagnosis.* Anal gland papillae poorly developed, bean-shaped, hardly visible and of the same colour as surrounding integument (Figure 5E); number of leg pairs overlapping between sexes: 26–27 in males and 27–29 in females (Table 1). *COI* and *12S rRNA* sequences as in specimens ME1-ME10 (Table 2). The species is characterised by 10 specific nucleotide positions in the *COI* sequence and a single position in the *12S rRNA* sequence (Table 3). Phylogenetic relationship as in Figure 6; intra- and interspecific distances as in Table S3.

*Description.* Body length after fixation 11.1–44.1 mm, width 1.7–3.9 mm, height 1.2–3.3 mm. Colour pattern in juveniles as in *E. adenocryptus* **sp. nov.** Antennae with 37 to 42 rings, 24 to 29 of which in antennal body and remaining 13 in antennal tip (including terminal button). Outer jaw blade with one principal and one accessory teeth (Figure S10E); inner jaw blade with one principal tooth, one or two accessory teeth and six to nine denticles (Figure S10F); accessory teeth and principal tooth of inner jaw blade similar in shape; accessory tooth either well-developed and with convex anterior and straight posterior faces or vestigial and thorn-shaped; asymmetric number of accessory teeth (either one or two) on inner jaws of left and right body sides in some specimens (as in *E. adenocryptus* **sp. nov.**). Remaining characters, shared with *E. diadenoproctus* **sp. nov.** and *E. adenocryptus* **sp. nov.**, as described above (see the Results section entitled “General anatomy of the specimens studied”).

*Remarks on habitat preference.* Specimens of *E. paurognostus* **sp. nov.** are found under rotten logs and in leaf litter close to watercourses. Juveniles occur within small pieces of rotten wood. Locals recorded the species in bean straw, which is used as a fertilizer in coffee plantations bordering the native forest remnant.

*Distribution. Epiperipatus paurognostus* **sp. nov.** has been recorded from the type locality, Mata do Eremitério, and from Rancho Primavera (localities III and IV in Figure 1A, B).

## Discussion

### Evidence of three new species of *Epiperipatus*

So far, only two onychophoran species, *Epiperipatus machadoi* (Oliveira & Wieloch, 2005) and *E. acacioi* (Marcus & Marcus, 1955), have been described from the Minas Gerais State of Brazil [4], although this region occupies an area about as large as France. However, a recent report of “*Peripatus* sp. 3” close to the type locality of *E. machadoi* suggests a higher number of species in this region than have been described thus far [5]. Our data indeed revealed three additional separate lineages occurring close to the type locality of *E. machadoi*. We recognise them as different species since the lineages are monophyletic in all our analyses and are delineated by their geographical locations. The molecular differences and the allopatric distribution indicate that these species do not hybridise and their recognition as species is therefore in accordance with both the biological and phylogenetic species concepts [17], [18].

Our statistical parsimony analyses revealed five separate networks, three of which correspond to the three allopatric species (*E. machadoi*, *E. diadenoproctus* **sp. nov.** and *E. paurognostus* **sp. nov.**), thus supporting the results of our phylogenetic analyses. In contrast, specimens of *E. adenocryptu*s **sp. nov.** fell apart in two unconnected haplotype networks, which is not in accordance with the assumption that the DNA sequences from single species typically stick together in a single haplotype network [19]. This result might be due to a low number of specimens sequenced and to numerous missing haplotypes in our analyses, which have caused the disruption of a single network in two separate subnetworks. Alternatively, *E. adenocryptus* **sp. nov.** might be a complex of two sympatric species. Including additional specimens and covering unsampled haplotypes in future analyses might clarify whether *E. adenocryptus* **sp. nov.** is a single species or a species complex.

All our specimens can be assigned to *Epiperipatus*, based on the presence of two anterior and one posterior foot papillae in most leg pairs, a low number of scale ranks in the basal pieces of dorsal primary papillae and the presence of crural papillae in two pre-genital leg pairs in males [1]–[4]. The three new species differ morphologically from *E. acacioi* and *E. machadoi* in that their males possess paired anal glands (also called “male accessory glands”), which open to the exterior on specialised papillae close to the anus ( =  anal gland papillae). Apart from the three new species, anal gland papillae have been described among representatives of *Epiperipatus* only in *E. biolleyi* (Bouvier, 1902) from Costa Rica and in *E. edwardsii* (Blanchard, 1847) from Sarare, Venezuela (Table S1). Given that *E. edwardsii* might be a species complex [5], the occurrence of anal gland papillae has to be clarified in specimens of *E. edwardsii* from other localities.

The paired anal glands and anal gland papillae are present in outgroup taxa, such as *Oroperipatus* and *Plicatoperipatus* from the neotropics and *Typhloperipatus* from South-East Asia [20]–[22] but are absent in *E. acacioi* and *E. machadoi*, which occur close to the localities of the three new species [4]. Clarifying whether the anal glands occur in other species of *Epiperipatus*, for which the corresponding data are currently missing (Table S1), will help understand the evolution of these structures and might provide a useful character for phylogenetic studies of the neotropical Peripatidae.

### Cryptic speciation and high endemism in the neotropical Peripatidae

The localised distributions of the three new species of *Epiperipatus* in a relatively small area of the Minas Gerais State of Brazil confirm Sampaio-Costa *et al.*'s [5] suggestion of high species diversity in the neotropical Peripatidae. In particular, *Epiperipatus* is according to our data one of the most speciose onychophoran genera, with numerous cryptic species still awaiting formal description. However, we caution that the monophyly of this genus has not been demonstrated yet and additional morphological (Table S1) and molecular data are required for a better understanding of species diversity and phylogeny of this onychophoran group.

The high endemism found in the three new species of *Epiperipatus* (Figure 1A, B) is not restricted to this taxon, but is a common phenomenon of Onychophora as it was also found in representatives of Peripatopsidae [7], [9], [12], [23]. One of the reasons for the high endemism of the onychophoran species might be their low dispersal ability since they are confined to microhabitats with high moisture levels (e.g., [24]). Therefore, the putative wide distribution of some species, such as *E. edwardsii*, *O. balzani* (Camerano, 1897) and *O. eisenii* (Wheeler, 1898), over hundreds or even thousands of kilometres [5], [25], is doubtful. The high endemism found amongst most other onychophoran species argues against the existence of species showing such a wide distribution. Previous reports of widely distributed species, thus, might be based on misidentified specimens and have to be reconsidered.

### Conclusion

In this paper, we give formal names and provide combined morphological and molecular diagnoses for three new species of *Epiperipatus*. This approach is uncommon among taxonomists, which is unfortunate because taxonomical studies based on morphological data alone apparently underestimate the cryptic diversity of Onychophora, in particular of the neotropical Peripatidae. Currently, a combination of morphological and molecular methods seems to be the best approach for delineating and identifying the onychophoran species, in particular of those lineages that show a low number of distinctive morphological characters. We believe that this approach will help handle the cryptic diversity and clarify the phylogeny and taxonomy of Onychophora in future studies. In addition, the potential value of the so-called “DNA barcodes” has to be considered for species identification in future studies of Onychophora as this method has proven useful in other animal groups (see, e.g., refs. [26], [27]). This will accelerate the slow pace, at which new onychophoran species are currently described, with only five formal species descriptions published in the last five years [28]–[32]. This is quite detrimental to studies of biological diversity because unnamed species are usually not taken into consideration for conservation programs.

## Materials and Methods

### Areas studied and collection of specimens

In addition to the two localities of *E. acacioi* and one of *E. machadoi*, four collecting sites were sampled (I–IV in Figure 1A, B): (I) RPPN Mata do Sossego (municipalities of Simonésia and Manhuaçu, 20°04′20″S, 42°04′12″W, 1150 m); (II) Córrego dos Ferreiras (municipality of Santa Bárbara do Leste, 42°06′46″W–19°58′59″S, 1050 m); (III) Mata do Eremitério (São José convent, municipality of Piedade de Caratinga, 42°05′22″W–19°45′33″S, 897 m); and (IV) Rancho Primavera (municipality of Piedade de Caratinga, 42°3′35.54″W–19°45′4.43″S, 830 m). All localities are areas of the fragmented Atlantic rain forest complex called Caratinga-Sossego.

Specimens were collected from leaf litter and from within or under rotten logs as described previously [4]. Most specimens from Mata do Sossego were found in human rubbish (roof clay tiles), placed in front of a researchers' accommodation near the border of the forest remnant. A total of 125 specimens were analysed (Table 4), including two single specimens from the Museu Nacional do Rio de Janeiro [MNRJ, National Museum of Rio de Janeiro] and the collection of the Departamento de Zoologia da Universidade Federal de Minas Gerais [DZUFMG, Department of Zoology of Universidade Federal de Minas Gerais]. All specimens were collected under the Brazilian federal license (ICMBio) number 10432/3.

**Table 4:** Number of specimens of new onychophoran species analysed from each locality.

Collecting site	Number of specimens used for	
	morphological analyses	molecular analyses
RPPN Mata do Sossego	62*	5
Córrego dos Ferreiras	34^**^	7
Mata do Eremitério	28	10
Rancho Primavera	1	-

*Including one specimen from the MNRJ collection. ^**^Including one specimen from the DZUFMG collection.

### Morphological analyses

The specimens were photographed *in vivo* and sacrificed using a piece of cotton soaked with ether and placed into a Petri dish. They were then analysed using an Olympus SZ61 stereomicroscope. In addition, a piece of dorsal integument and the fifth right leg from each specimen were fixed, handled and analysed in a Quanta 200-FEG-FEI-2006 Scanning Electron Microscope (FEI, Hillsboro, Oregon, USA) as described previously [4]. Additional data were obtained from the type series of (1) *Peripatus heloisae* Carvalho, 1941, (2) *Epiperipatus acacioi* and (3) *E. machadoi*, held in the MNRJ, the Museu de Zoologia da Universidade de São Paulo [MZUSP, Museum of Zoology of Universidade de São Paulo] and the DZUFMG, respectively. The terminology of morphological terms was used according to Oliveira *et al.* [4].

### Molecular and phylogenetic analyses

Tissue samples from 22 specimens from different localities were used for molecular studies (Table 4). In addition, we included four specimens of *E. machadoi* from RPPN Feliciano Miguel Abdala in our analyses for comparison since this species occurs close to the type localities of the three new species described herein (Figure 1A, B; Table 4). *Epiperipatus biolleyi* was selected as an outgroup since it was the only species of the genus, for which all required molecular data were available [33]; GenBank accession number: DQ666064).

The samples were preserved in ethanol. The genomic DNA was extracted from body pieces (∼25 mg) using the DNeasy Tissue Kit (Qiagen, Hilden, Germany) according to the manufacturer's protocol. DNA sequences of the mitochondrial *COI* gene were amplified using the specific primers COI5584 (5′-TGTGACTGGTCATGCATTTGT-3′) and COI6174 (5′-GAAACTATTCCAAAGCCAGGAA-3′), designed for this study using the *COI* sequence of *E. biolleyi*. The sequences of the mitochondrial *12S rRNA* gene were amplified using the primers SR-J-14233 and SR-N-14588 from Simon *et al.* [34]. The *COI* and *12S rRNA* loci were selected because they show numerous variable sites and were used successfully in studies of genetic variation and cryptic speciation in Peripatopsidae [8], [9], [11], [12], [14].

PCR amplifications were performed in 20 µl reaction volumes containing 40 ng genomic DNA, Buffer 1B (Phoneutria®, Belo Horizonte, Brazil: 1.5 mM MgCl_2_, 10 mM Tris-HCl, 50 mM KCl, 0.1% Triton X-100), 0.8 µM dNTPs, 0.3 µM primers, 1% bovine serum albumin (BSA) and 1 unit *Taq* polymerase (Phoneutria®). After an initial denaturing step for 5 min at 94°C, the PCR conditions for the *COI* and *12S rRNA* fragments followed a standard three-step protocol, with 27 cycles of (1) denaturing for 45 s at 94°C, (2) annealing for 45 s at 56°C (*COI* primers) or 54°C (*12S rRNA* primers), and (3) extension for 1 min at 72°C, followed by a final extension step for 5 min at 72°C. The PCR products were purified using a solution of 20% polyethylene-glycol (PEG 8000) and 2.5M NaCl according to Sambrook *et al.* [35]. After purification, the PCR products were sequenced in both directions using the BigDye Terminator Kit v3 (Applied Biosystems, Foster City, USA) and an ABI3100® automated sequencer (Applied Biosystems, Foster City, USA). The sequences were assembled and checked for quality using Phred v.0.20425 [36], [37] and Phrap v.0.990319 [38] and the assembled chromatograms were verified and edited using Consed 12.0 [39]. The sequences were deposited in the GenBank database (Table 2).

The obtained sequences were aligned using the Clustal W algorithm implemented in MEGA 4.1 [40]. This software was also used for calculating the intraspecific and interspecific genetic distances using the Kimura 2-parameter (K2P) model. Modeltest 3.7 [41] was used to select the best-fit model of sequence evolution. The selected model for the *COI* data set was the General Time Reversible Model (GTR + I + Γ) with gamma distributed (Γ) rates α = 0.6886 and a proportion of invariant sites I = 0.3536. For the *12S rRNA* data set, the model GTR + Γ was selected with α = 0.2944 and I = 0. For the combined data set, the selected model was GTR + I + Γ, with α = 0.8048 and I = 0.4049.

For phylogenetic analyses, NJ, MP, ML and BI were used. The NJ analyses were conducted using PAUP*4.0b10 [42] and the ML distances were determined using Modeltest 3.7 [41]. The support for each clade was assessed by 1,000 bootstrap replicates [42]. The MP analyses were implemented using PAUP*4.0b10 [42], including 100 replicates of random sequence addition with tree bisection and reconnection (TBR) branch swapping. The support for each clade was assessed by 1,000 bootstrap replicates [43] and by estimating Bremer support values (decay index-DI) of the strict consensus tree. Unambiguous synapomorphies were identified after performing the MP analyses and used as diagnostic molecular characters for each species. The TNT software was used for estimating Bremer support values and identifying unambiguous synapomorphies [44]. For the ML inference analyses, the PhyML software [45] was implemented using the selected model and support of clades assessed by bootstrap analyses with 1,000 replicates. The BI analysis was performed with four Markov chain Monte Carlo chains, which run simultaneously for 10,000,000 generations, with trees sampled every 100 generations for a total of 100,000 trees. Posterior probabilities were calculated based on trees retained after log-likelihood values had stabilised. All BI analyses were performed with MrBayes v3.0b4 [46].

To calculate the haplotype networks, we performed a statistic parsimony analysis of the *COI* sequences from all 22 specimens sequenced and four additional specimens of *E. machadoi* using the TCS v1.21 software [47]. The connection limit excluding the homoplastic changes was set to 95% according to Hart & Sunday [19], whose statistic analyses of empirical data have shown that alignments of DNA sequences typically fall apart into separate networks corresponding to Linnean species. This suggests that network parsimony analyses are useful for species detection using molecular data, in particular the mitochondrial DNA data sets [19], [48].

### Taxonomic Registration and Digital Archiving

The electronic version of this document does not represent a published work according to the International Code of Zoological Nomenclature (ICZN), and hence the nomenclatural acts contained in the electronic version are not available under that Code from the electronic edition. Therefore, a separate edition of this document was produced by a method that assures numerous identical and durable copies, and those copies were simultaneously obtainable (from the publication date noted on the first page of this article) for the purpose of providing a public and permanent scientific record, in accordance with Article 8.1 of the Code. The separate print-only edition is available on request from PLoS by sending a request to PLoS ONE, Public Library of Science, 1160 Battery Street, Suite 100, San Francisco, CA 94111, USA along with a check for $10 (to cover printing and postage) payable to "Public Library of Science".

The online version of the article is archived and available from the following digital repositories: PubMedCentral (www.pubmedcentral.nih.gov/), and LOCKSS (http://www.lockss.org/lockss/). In addition, this published work and the nomenclatural acts it contains have been registered in ZooBank, the proposed online registration system for the ICZN. The ZooBank LSIDs (Life Science Identifiers) can be resolved and the associated information viewed through any standard web browser by appending the LSID to the prefix "http://zoobank.org/". The ZooBank LSID for this publication is: urn:lsid:zoobank.org:pub:E0156AE2-CD2D-415B-94C8-6278B4A12FD2.

## Supporting Information

Figure S1 **Body colour pattern in living specimens of** ***E. adenocryptus*** **sp. nov. (A, B) and** ***E. paurognostus*** **sp. nov. (C, D).** A, C, Specimens in dorsal view. B, D, Specimens in ventral view. Arrows indicate the ventral organs. Abbreviation: mo, mouth.
(TIF)

Figure S2 **Arrangement of accessory (white dots) and primary papillae (black circles) along dorsal midline in the new onychophoran species.** Female paratypes of *E. diadenoproctus* **sp. nov.** (A, B), *E. adenocryptus* **sp. nov.** (C, D) and *E. paurognostus* **sp. nov.** (E, F).
(TIF)

Figure S3 **Alignment of nucleotide sequences of the mitochondrial *COI* gene in the sampled taxa.** **Dots indicate similar bases between the specimens studied and** *Epipepipatus machadoi* **(FM1). The first nucleotide corresponds to the first codon position. Abbreviations as per** **Table 2**.
(TIF)

Figure S4 **Alignment of amino acid sequences inferred from** ***COI*** **nucleotide sequences.** Dots indicate similar amino acids between the specimens studied and *Epipepipatus machadoi* (FM1). Abbreviations as per Table 2.
(TIF)

Figure S5 **Alignment of nucleotide sequences of the mitochondrial *12S rRNA* gene in the sampled taxa.** **Dots indicate similar bases between the specimens studied and** *Epipepipatus machadoi* **(FM1). Abbreviations as per** **Table 2**.
(TIF)

Figure S6 **Maximum Parsimony (MP) topology for combined mitochondrial data sets (** ***COI + 12S rRNA*** **) amongst** ***Epiperipatus*** **specimens studied.** *Epiperipatus biolleyi* was used as an outgroup. Upper numbers at each node represent bootstrap support values, lower numbers are absolute and relative Bremer support values. Abbreviations as per Table 2.
(TIF)

Figure S7 **Bayesian Inference (BI) topology for combined mitochondrial data sets (** ***COI + 12S rRNA*** **) amongst** ***Epiperipatus*** **specimens studied.** *Epiperipatus biolleyi* was used as an outgroup. Numbers at each node are Bayesian posterior probabilities. Scale bar represents genetic distance (substitutions per site). Abbreviations as per Table 2.
(TIF)

Figure S8 **Neighbor-Joining (NJ) topology for combined mitochondrial data sets (** ***COI + 12S rRNA*** **) amongst** ***Epiperipatus*** **specimens studied.** *Epiperipatus biolleyi* was used as an outgroup. Numbers at each node are bootstrap support values. Scale bar represents genetic distance (substitutions per site). Abbreviations as per Table 2.
(TIF)

Figure S9 **Maximum Likelihood (ML) topology for combined mitochondrial data sets (** ***COI*** **+** ***12S rRNA*** **) amongst** ***Epiperipatus*** **specimens studied.** *Epiperipatus biolleyi* was used as an outgroup. Numbers at each node are bootstrap support values. Scale bar represents genetic distance (substitutions per site). Abbreviations as per Table 2.
(TIF)

Figure S10 **Structure of outer and inner jaw blades in the new onychophoran species.** Light micrographs. A, B, *Epiperipatus diadenoproctus* **sp. nov.** C, D, *E. adenocryptus* **sp. nov.** E, F, *E. paurognostus* **sp. nov.** Anterior is up, ventral is left in all images. A, Outer jaw blade with a principal tooth and two accessory teeth. B, Inner jaw blade with a principal tooth, two accessory teeth and ten denticles. C, Outer jaw blade with a principal tooth and one accessory tooth. D, Inner jaw blade with a principal tooth, two accessory teeth and six denticles. E, Outer jaw blade with a principal tooth and one accessory tooth. F, Inner jaw blade with a principal tooth, two accessory teeth, and seven denticles. Abbreviations: at, accessory tooth/teeth; dt, denticles; pt, principal tooth.
(TIF)

Table S1 **Comparison of anatomical features in** ***Epiperipatus*** **species described thus far.**
(DOC)

Table S2 **Summary of statistics for sequence data.**
(DOC)

Table S3 **Average genetic distances within and between onychophoran taxa (** ***COI*** **+** ***12S rRNA*** **) according to the Kimura 2-parameter model.**
(DOC)
