# Supplementary figures and images for: Cryptic Speciation in Brazilian Epiperipatus (Onychophora: Peripatidae) Reveals an Underestimated Diversity among the Peripatid Velvet Worms
Source: PLoS One. 2011 Jun 10;6(6):e19973. doi: 10.1371/journal.pone.0019973 (PMC3112143; doi:10.1371/journal.pone.0019973)

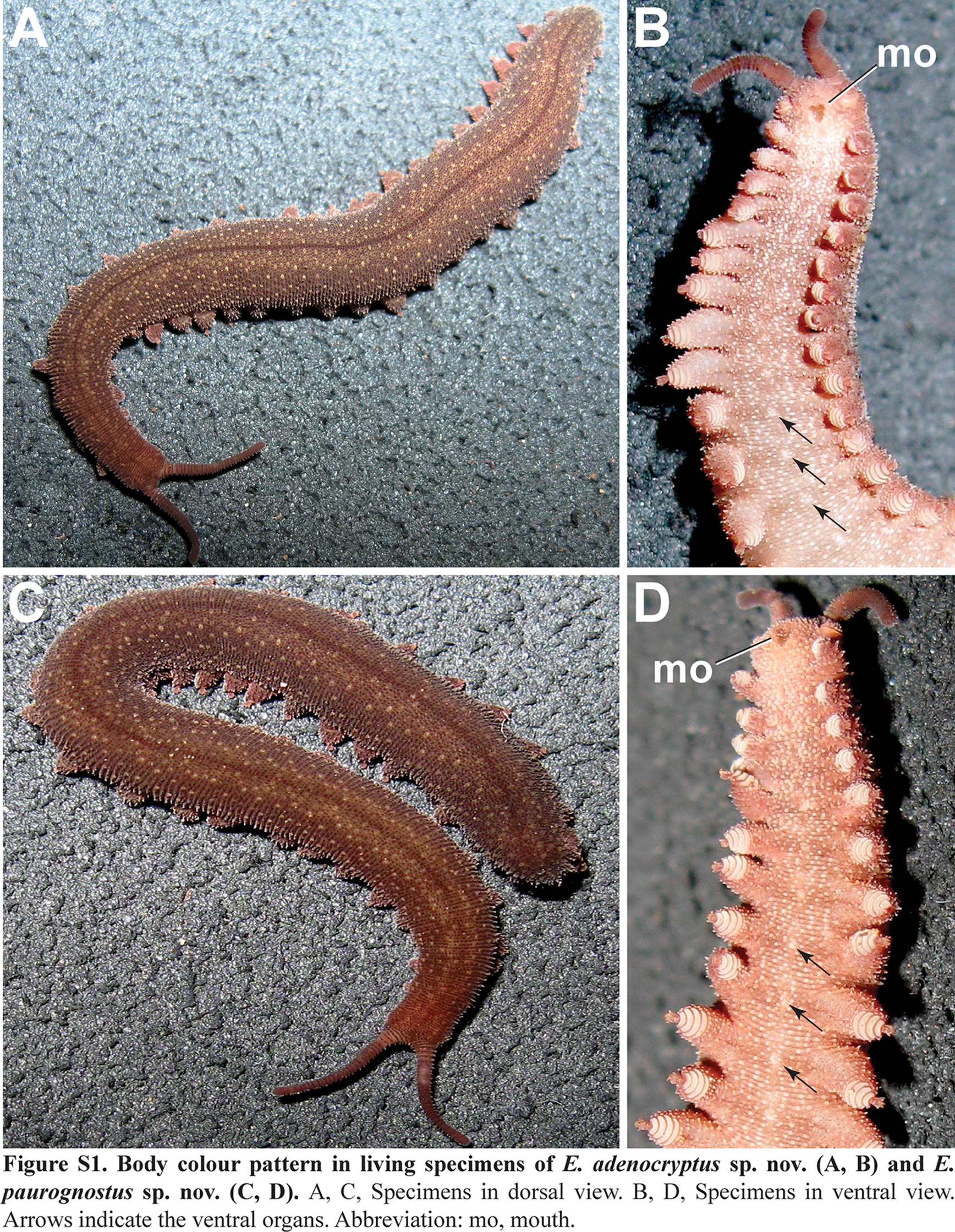

Supplement: Figure S1 — Body colour pattern in living specimens of E. adenocryptus sp. nov. (A, B) and E. paurognostus sp. nov. (C, D). A, C, Specimens in dorsal view. B, D, Specimens in ventral view. Arrows indicate the ventral organs. Abbreviation: mo, mouth. (TIF) [file pone.0019973.s001.tif]

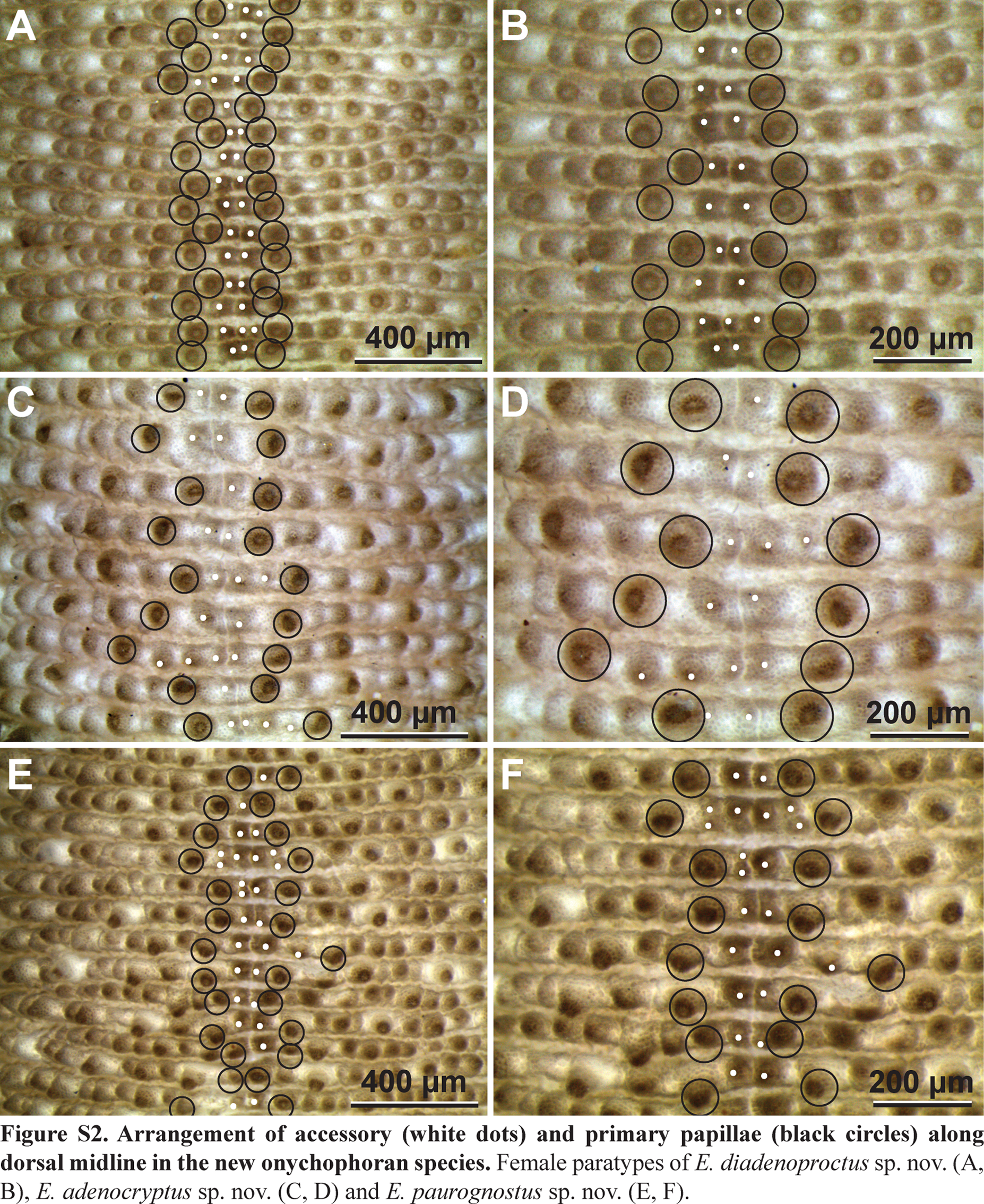

Supplement: Figure S2 — Arrangement of accessory (white dots) and primary papillae (black circles) along dorsal midline in the new onychophoran species. Female paratypes of E. diadenoproctus sp. nov. (A, B), E. adenocryptus sp. nov. (C, D) and E. paurognostus sp. nov. (E, F). (TIF) [file pone.0019973.s002.tif]

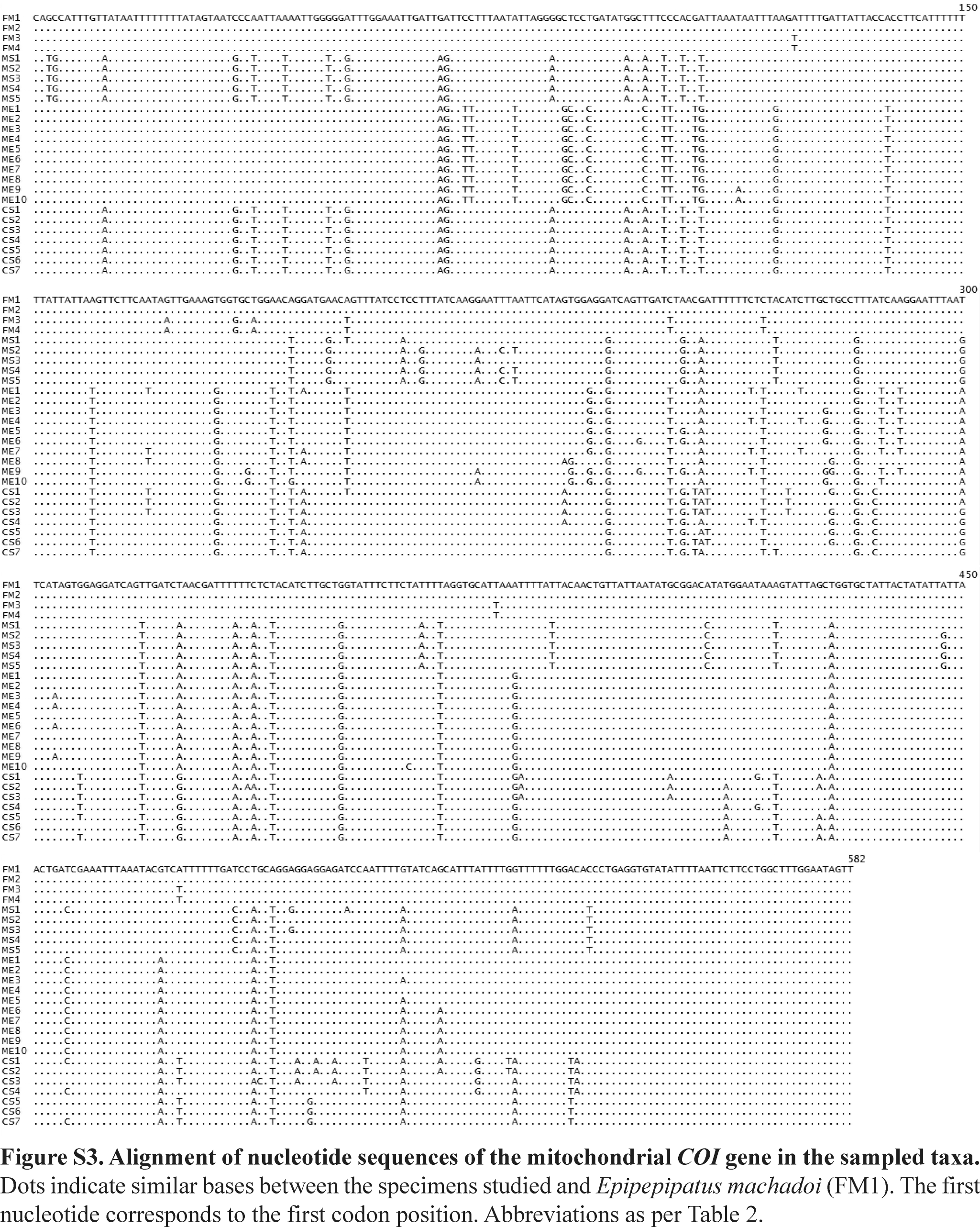

Supplement: Figure S3 — Alignment of nucleotide sequences of the mitochondrial COI gene in the sampled taxa. Dots indicate similar bases between the specimens studied and Epipepipatus machadoi (FM1). The first nucleotide corresponds to the first codon position. Abbreviations as per Table 2 . (TIF) [file pone.0019973.s003.tif]

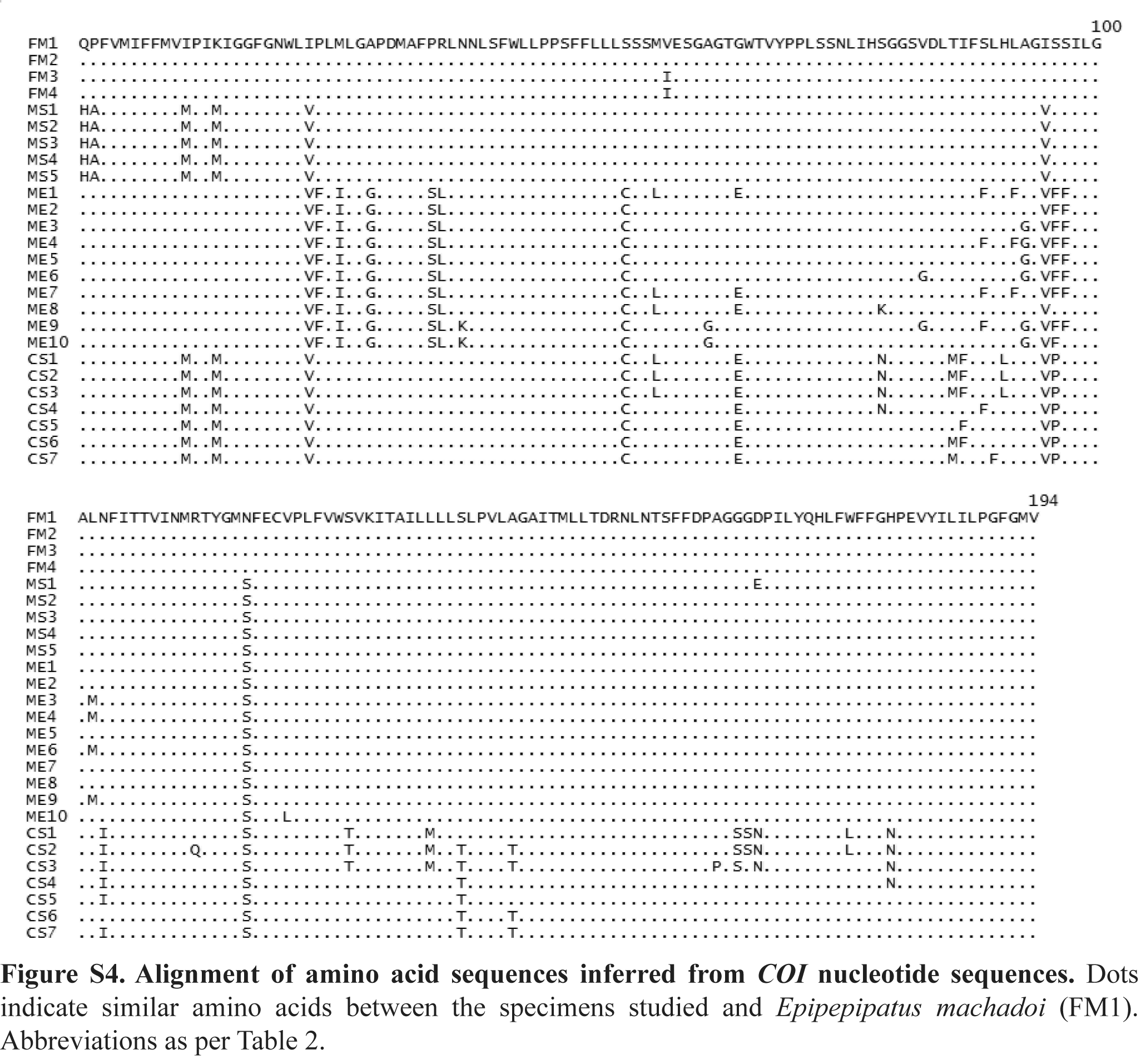

Supplement: Figure S4 — Alignment of amino acid sequences inferred from COI nucleotide sequences. Dots indicate similar amino acids between the specimens studied and Epipepipatus machadoi (FM1). Abbreviations as per Table 2. (TIF) [file pone.0019973.s004.tif]

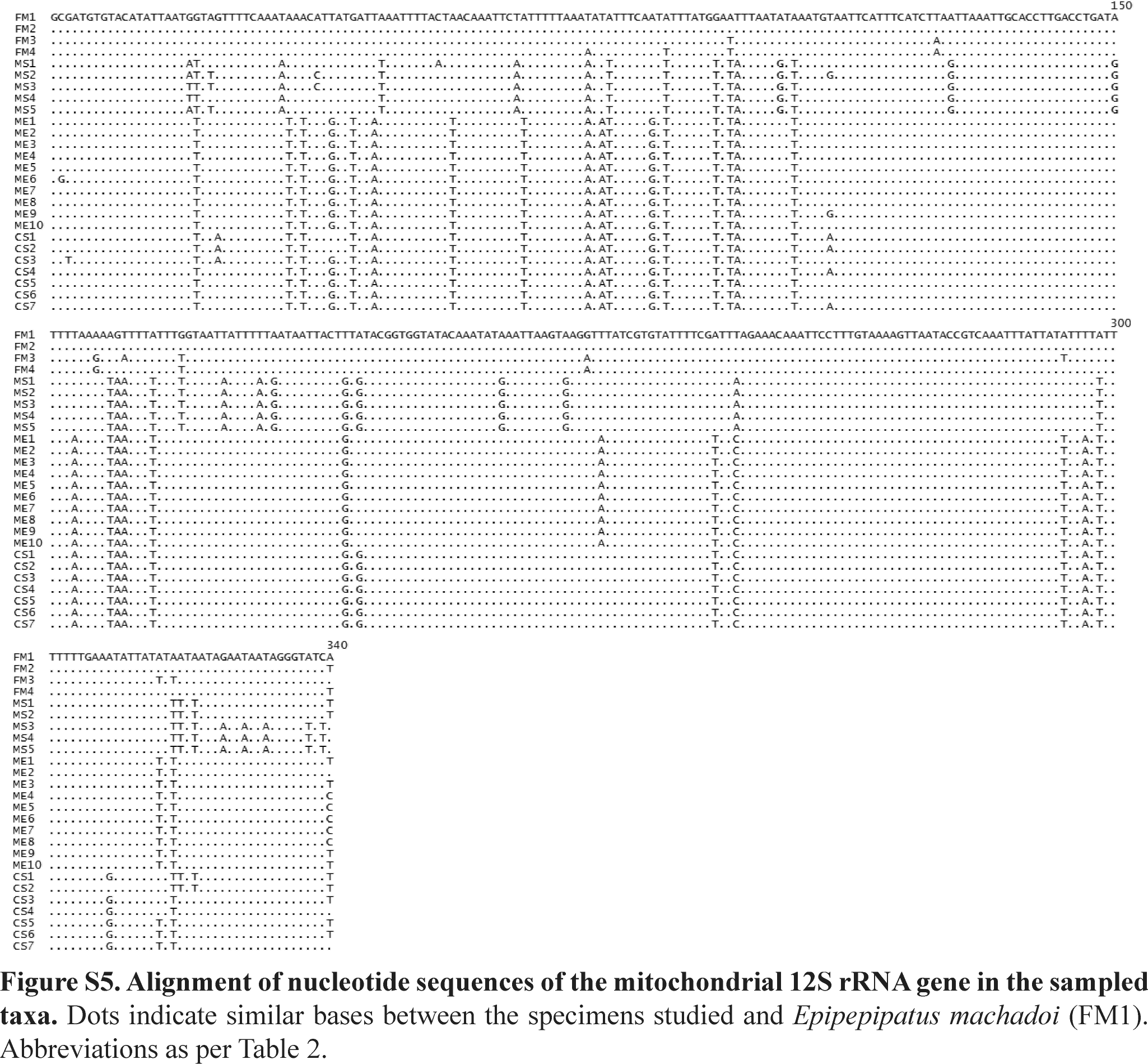

Supplement: Figure S5 — Alignment of nucleotide sequences of the mitochondrial 12S rRNA gene in the sampled taxa. Dots indicate similar bases between the specimens studied and Epipepipatus machadoi (FM1). Abbreviations as per Table 2 . (TIF) [file pone.0019973.s005.tif]

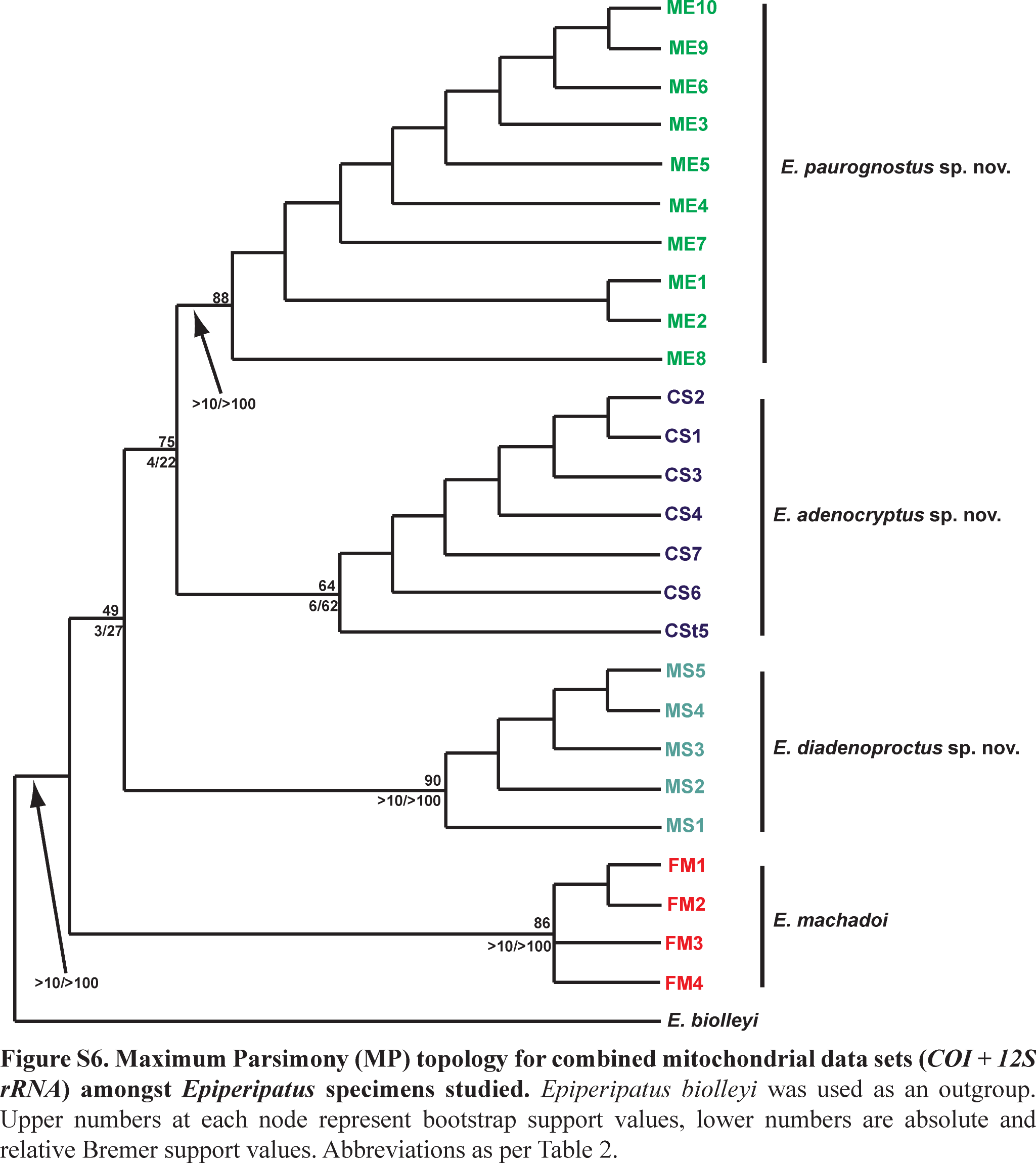

Supplement: Figure S6 — Maximum Parsimony (MP) topology for combined mitochondrial data sets ( COI + 12S rRNA ) amongst Epiperipatus specimens studied. Epiperipatus biolleyi was used as an outgroup. Upper numbers at each node represent bootstrap support values, lower numbers are absolute and relative Bremer support values. Abbreviations as per Table 2. (TIF) [file pone.0019973.s006.tif]

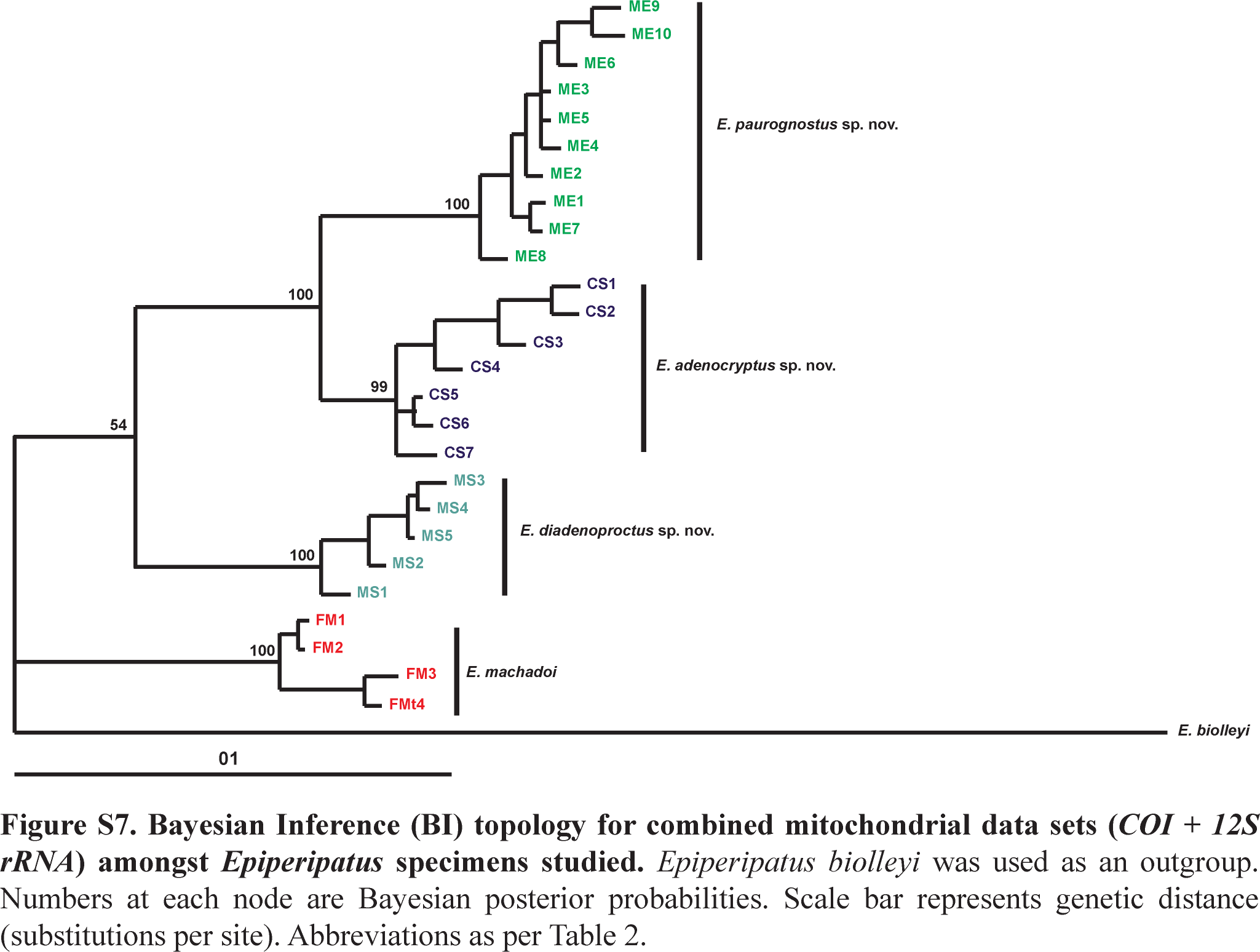

Supplement: Figure S7 — Bayesian Inference (BI) topology for combined mitochondrial data sets ( COI + 12S rRNA ) amongst Epiperipatus specimens studied. Epiperipatus biolleyi was used as an outgroup. Numbers at each node are Bayesian posterior probabilities. Scale bar represents genetic distance (substitutions per site). Abbreviations as per Table 2. (TIF) [file pone.0019973.s007.tif]

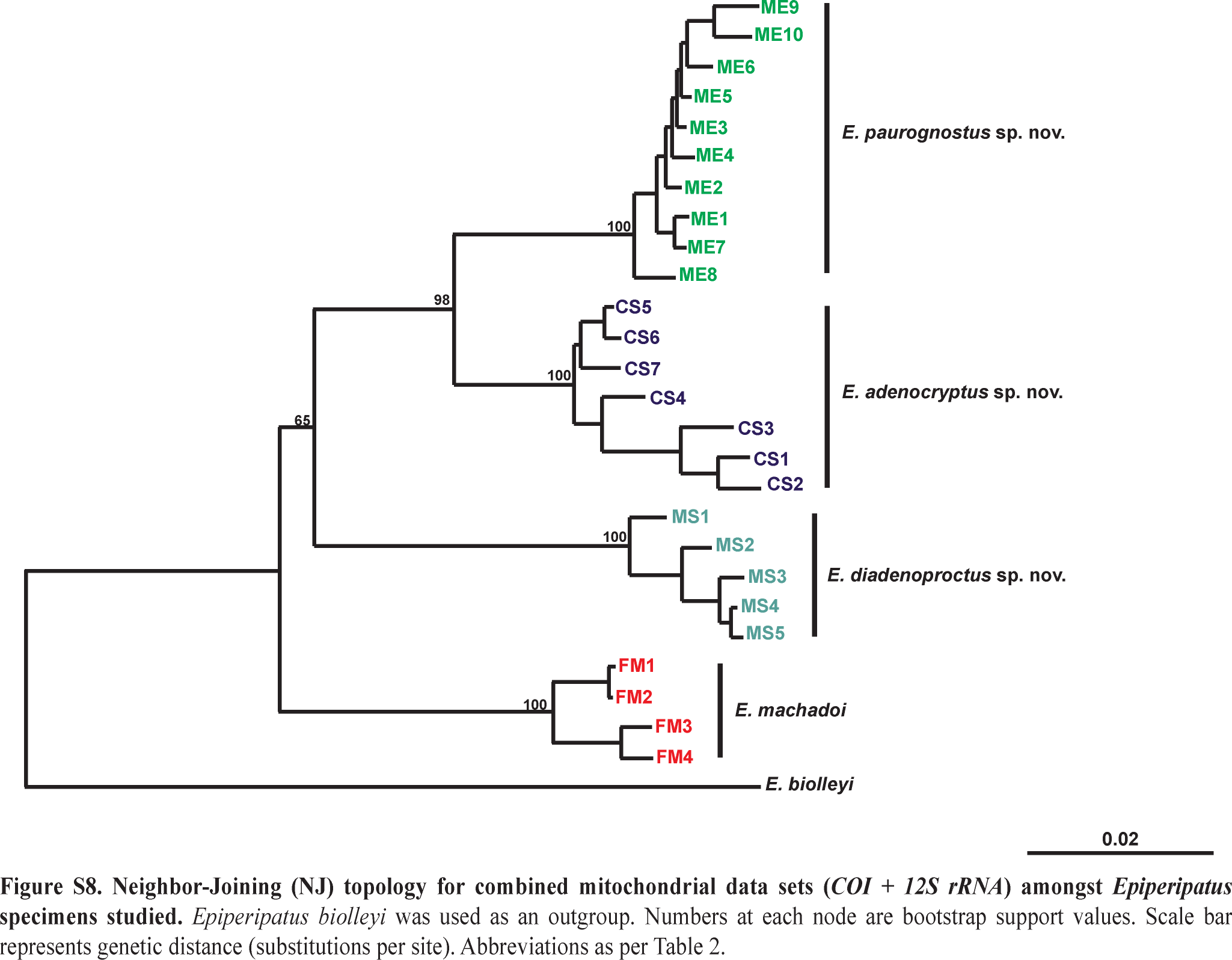

Supplement: Figure S8 — Neighbor-Joining (NJ) topology for combined mitochondrial data sets ( COI + 12S rRNA ) amongst Epiperipatus specimens studied. Epiperipatus biolleyi was used as an outgroup. Numbers at each node are bootstrap support values. Scale bar represents genetic distance (substitutions per site). Abbreviations as per Table 2. (TIF) [file pone.0019973.s008.tif]

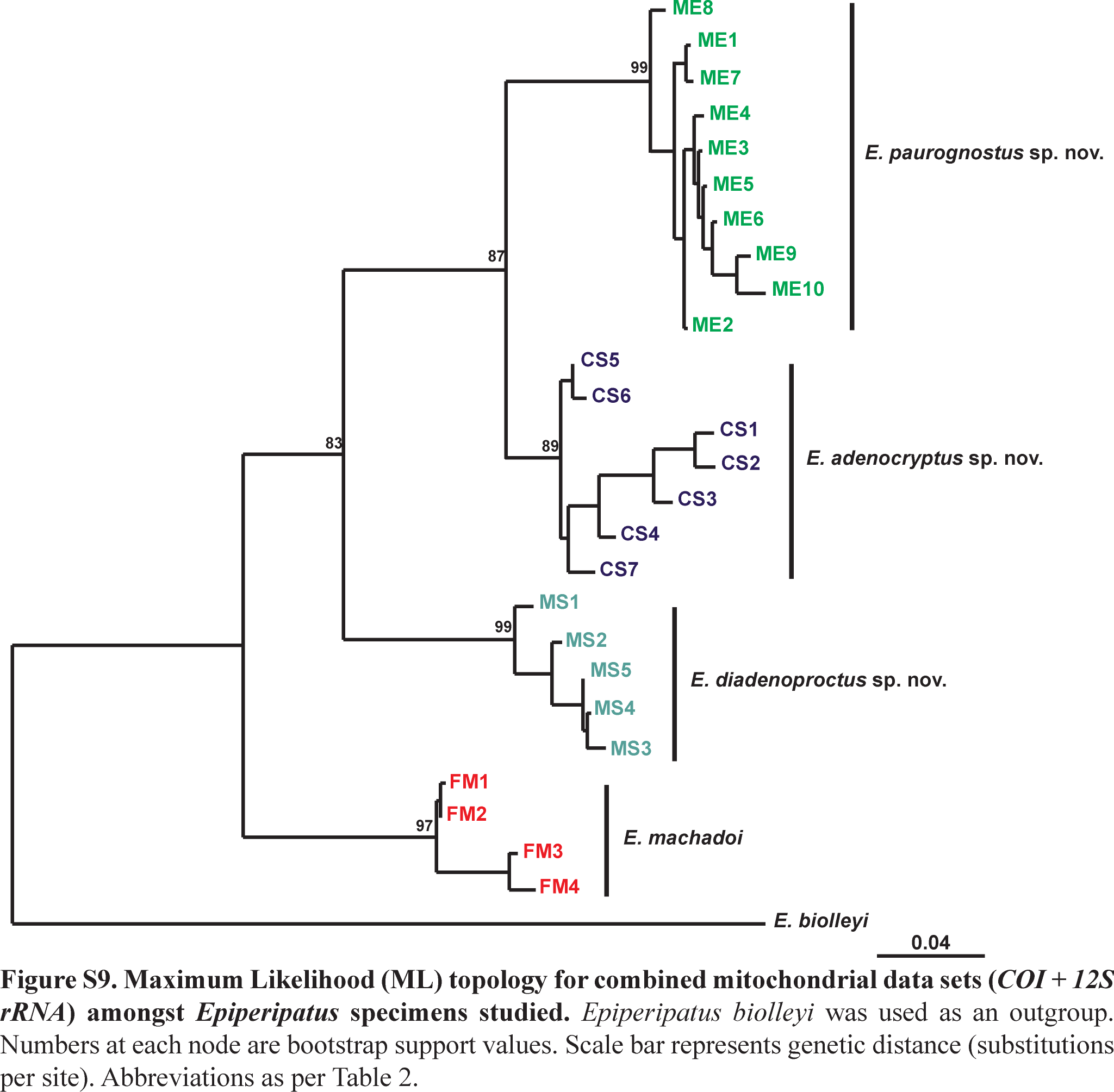

Supplement: Figure S9 — Maximum Likelihood (ML) topology for combined mitochondrial data sets ( COI + 12S rRNA ) amongst Epiperipatus specimens studied. Epiperipatus biolleyi was used as an outgroup. Numbers at each node are bootstrap support values. Scale bar represents genetic distance (substitutions per site). Abbreviations as per Table 2. (TIF) [file pone.0019973.s009.tif]

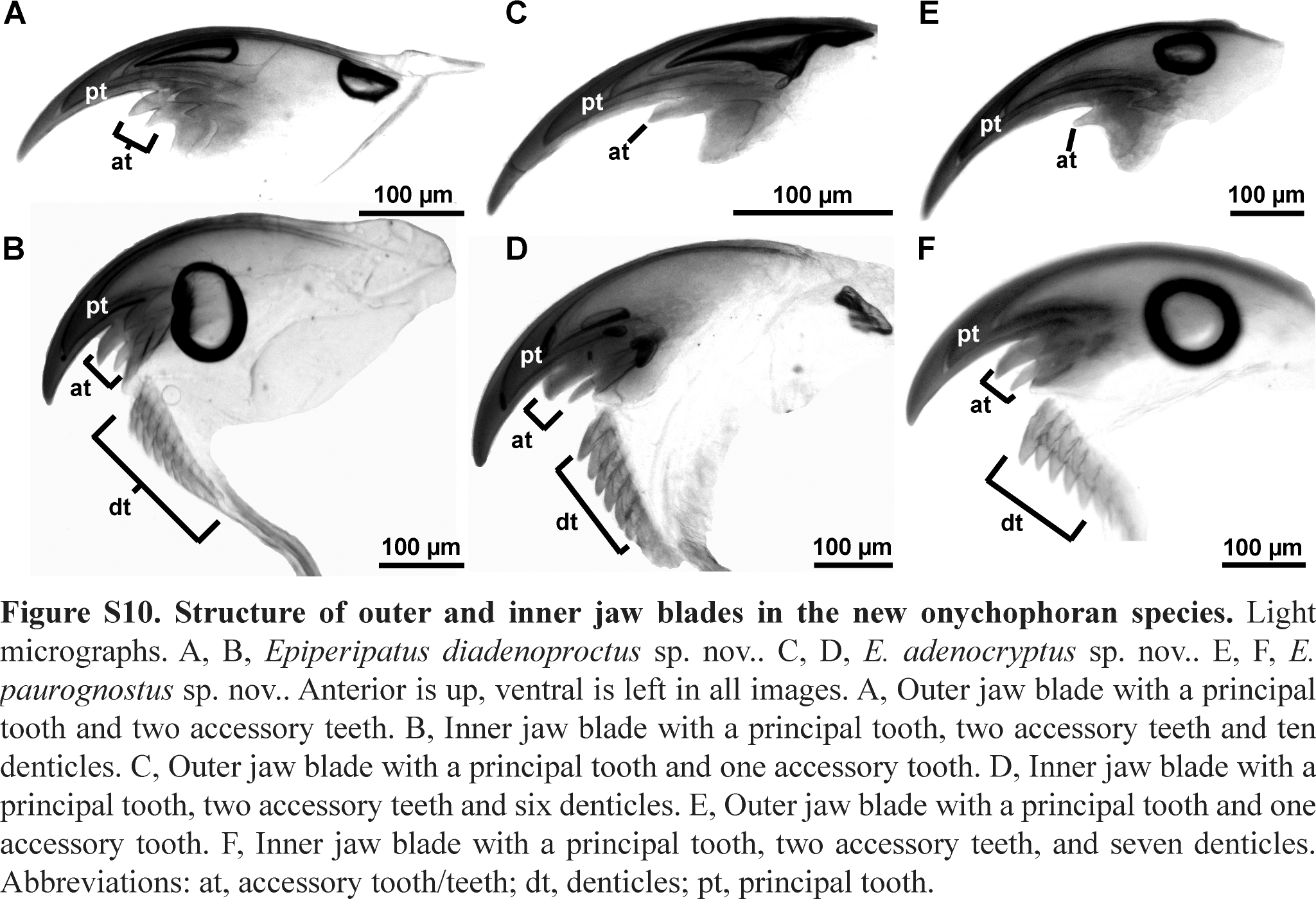

Supplement: Figure S10 — Structure of outer and inner jaw blades in the new onychophoran species. Light micrographs. A, B, Epiperipatus diadenoproctus sp. nov. C, D, E. adenocryptus sp. nov. E, F, E. paurognostus sp. nov. Anterior is up, ventral is left in all images. A, Outer jaw blade with a principal tooth and two accessory teeth. B, Inner jaw blade with a principal tooth, two accessory teeth and ten denticles. C, Outer jaw blade with a principal tooth and one accessory tooth. D, Inner jaw blade with a principal tooth, two accessory teeth and six denticles. E, Outer jaw blade with a principal tooth and one accessory tooth. F, Inner jaw blade with a principal tooth, two accessory teeth, and seven denticles. Abbreviations: at, accessory tooth/teeth; dt, denticles; pt, principal tooth. (TIF) [file pone.0019973.s010.tif]
