# Supplementary material for: Cryptic Speciation in Brazilian Epiperipatus (Onychophora: Peripatidae) Reveals an Underestimated Diversity among the Peripatid Velvet Worms
Source: PLoS One. 2011 Jun 10;6(6):e19973. doi: 10.1371/journal.pone.0019973 (PMC3112143; doi:10.1371/journal.pone.0019973)
Supplement: Table S1 — Comparison of anatomical features in Epiperipatus species described thus far. (DOC) [file pone.0019973.s011.doc]

**Table S1.** Comparison of anatomical features in *Epiperipatus* species.

| **Species** | **Dorsal colour pattern of integument** | **Anastomosing or incomplete dorsal plicae absent (a) or present (p)** | **Fourth spinous pad well-developed (w) or reduced in size (r)** | **Vestigial/fragmented fifth spinous pad absent (a) or present (p)** | **Nephridial tubercle attached to (t) or detached from the third spinous pad (d)** | **Number of antennal rings** | **Number of leg pairs in males** | **Number of leg pairs in females** | **Number of scale ranks in apical piece^1^** | **Number of scale ranks in basal piece^2^** | **Apical piece symmetrical (s) or asymmetrical (n)** | **Anal gland papillae absent (a) or present (p)** | **Coxal vesicles absent (a) or present (p)** | **References** |
| --- | --- | --- | --- | --- | --- | --- | --- | --- | --- | --- | --- | --- | --- | --- |
| ***E. acacioi*** | dark-purple with bilaterally symmetrical repeated bracket-like pattern along dorsal midline | p | w | p | t | 33–42 | 24–27 | 26–29 | 3/2 | 5/5–6 | n | a | p | Marcus & Marcus (1955); Froehlich (1968, pp. 169-170); Oliveira *et al.* (2010, pp. 21-25) |
| ***E. adenocryptus* sp. nov.** | brown with repeated concave light-brown arcs on each side of dorsal midline forming circles | p | w | p | t/d | 38–43 | 26–27 | 28–30 | 3–4/2 | 4–6/7–8 | n | p | p | present study |
| ***E. barbadensis*** | homogeneously dull dark-brown or reddish-brown | p | w | p | d | 42 | ? | 31 | 3 | (~8)^3^ | s | ? | ? | Froehlich (1962); Read (1988b, pp. 236-237, 239) |
| ***E. barbouri*** | dark-purplish (slate-like), almost black, without any pattern | p | w | ? | t | ? | ? | 30–32 | 2 or 3 | (~12)^3^ | s | ? | ? | Brues (1911); Read (1988b, pp. 238-240) |
| ***E. biolleyi*** | red, without any pattern | p | w | p | d | ? | 26–28 | 30 | 3/2 | (6–7)^3^ | n | p | a | Bouvier (1902; 1905, pp. 321-326); G. Mayer (unpublished data) |
| ***E. brasiliensis*** | yellowish-brown, greyish or pallid with bright dilatations along dorsal midline | a | w | a | d | 40–44 | 29 | 31–33 | 3 | ? | s | a | ? | Bouvier (1899, 1905, pp. 269-275); Froehlich (1968, pp. 169); Read (1988a, pp. 197, 214) |
| ***E. broadwayi*** | dark-brown with bilateral light-brown triangles along dorsal midline (see Read 1988b, p. 245) | p | w | ? | d | ? | (29–34)^4^ | | 2, or probably 3 | (5–7)^3^ | s | ? | ? | Clark (1913, pp. 254-255); Read (1988b, pp. 244-245, 247) |
| ***E. cratensis*** | purple to white | a | w | ? | ? | 31–36 | 33 | 34 | (3 or 4)^3^ | (5–7)^3, 5^ | s^5^ | ? | p | Brito *et al.* (2010) |
| ***E. diadenoproctus* sp. nov.** | brown with repeated concave light-brown arcs on each side of dorsal midline forming circles | p | w | p | t/d | 38–43 | 26–28 | 29–30 | 3–4/2 | 5–6/7 | n | p | p | present study |
| ***E. edwardsii*** | similar to *E. broadwayi* (see Read 1988b, p. 251) | p | w | a | d | 45–48 | 26–29 | 29–34 | 3 | (~9)^3^ | s | (p/?)^6^ | p | Blanchard (1847); Bouvier (1905, pp. 301-315); Froehlich (1968); Read (1988a, p. 203; 1988b, pp. 249-251, 253) |
| ***E. evansi*** | purplish dark-brown with bright triangles on each side of dorsal midline forming repeated diamonds | p | w | ? | t | ? | ? | 28 | 3 | (~6)^3^ | s | ? | ? | Bouvier (1904, 1905, pp. 285-289); Read (1988a, pp. 194-196) |
| ***E. hilkae*** | dark-brown with light reddish-brown papillae forming repeated hexagons along dorsal midline | p | r | a | d | ? | 25–27 | 28–29 | 2 or 3 | (4–5)^3, 5^ | (s)^5^ | ? | ? | Morera-Brenes & Monge-Nájera, (1990) |
| ***E. imthurni*** | light-orange or yellowish-brown, without any pattern | p | w | ? | d | ? | −^7^ | 29–32 | 3 | (~12)^3^ | s | a | ? | Sclater (1888); Bouvier (1905, pp. 275-285); Read (1988a, p. 204; 1988b, pp. 240-243) |
| ***E. isthmicola*** | uniformly dark-brown, almost black, without any pattern | p | r | a | d | ? | 26 | 29–32 | 3 | (~14)^3^ | s | a | a | Bouvier (1902; 1905, pp. 329-333); Read (1988a, p. 207) |
| ***E. lewisi*** | from grey to intense dark reddish-brown | p (as in *E. edwardsii*) | w | ? | t | ? | (34–36)^4^ | | ? | ? | ? | ? | ? | Arnett (1961) |
| ***E. machadoi*** | dark-brown with repeated concave light-brown arcs on each side of dorsal midline forming circles | p | w | p | t | 38–49 | 27–29 | 29–31 | 3/2 | 5–7/8 | n | a | p | Oliveira & Wieloch (2005); Oliveira *et al.* (2010, pp. 25-29) |
| ***E. nicaraguensis*** | uniform dark-brown, almost black, without any pattern | p | r | a | d | ? | ? | 32 | ? | ? | ? | ? | ? | Bouvier (1900; 1905, pp. 326-329) |
| ***E. paurognostus* sp. nov.** | brown, with repeated concave light-brown arcs on each side of dorsal midline forming circles | p | w | p | t/d | 37–42 | 26–27 | 27–29 | 3–4/2 | 5–6/7–8 | n | p | p | present study |
| ***E. simoni*** | uniform dark-brown, without any pattern | p | w | a | t | ? | ? | 28–32 | ? | ? | ? | ? | ? | Bouvier (1898; 1905, pp. 315-320) |
| ***E. torrealbai*** | dark reddish-brown, without any pattern | p | w | ? | t | ? | ? | 31 | ? | ? | ? | ? | ? | Scorza (1953) |
| ***E. trinidadensis*** | similar to *E. broadwayi*, but less intense (see Read 1988b: 248) | p | w | ? | t | ? | 27–30 | 28–31 | 3 | (6–7)^3^ | s | ? | ? | Sedgwick (1888); Bouvier (1905, pp. 289-301); Read (1988b, pp. 246-249) |
| ***E. tucupi*** | ? | a | w | p | t | 43–44 | ? | 34–35 | 4 | ? | s | ? | ? | Froehlich (1968); Read (1988a, p. 198) |
| ***E. vespucci*** | dorsal integument dark, with a complex colour pattern (see Brues 1914, p. 376); legs lighter than dorsal jntegument | p | w | ? | d | ? | 30 | 33–34 | ? | ? | ? | ? | ? | Brues (1914) |

Question marks indicate missing data. Imprecise information from the literature is indicated by a “~”. ^1^Anterior and posterior numbers of scale ranks are separated by a slash. ^2^Laterial/median and anterior/posterior numbers of scale ranks are separated by a slash. ^3^Lateral/median and anterior/posterior numbers of scale ranks are not distinguished in the literature. ^4^Numbers of leg pairs have not been reported for each sex. ^5^Based on scanning electron micrographs provided. ^6^Present in specimens from Sarare, Venezuela, but unknown in specimens from other localities. ^7^The males are unknown in *E. imthurni*, which is most likely a parthenogenetic species (Read, 1988b: 243).

**References**

Arnett, R. H. (1961). The Onychophora of Jamaica. *Entomological News*, 72, 213–220.

Blanchard, E. (1847). Recherches sur l'organisation des Vers. *Annales des Sciences Naturelles [3e Série]*, 8, 119–149.

Bouvier, E. L. (1898). Sur les caractères externes des Péripates. *Proceedings of the International Congress of Zoology*, 4, 269–271.

Bouvier E. L. (1899). Nouvelles observations sur les Péripates américains. *Comptes Rendus Hebdomadaires des Seances de l'Academie des Sciences*, 129, 1029–1031.

Bouvier, E. L. (1900). Nouveau Péripate des environs de Rio-de-Janeiro. *Bulletin de la Société Entomologique de France*, [1900], 66–68.

Bouvier, E. L. (1902). *Peripatus biolleyi*, Onychophore nouveau de Costa-Rica. *Bulletin de la Société Entomologique de France*, 16, 258–259.

Bouvier, E. L. (1904). Les oeufs des Onychophores. *Museum d’Histoire Naturelle de Lyon. Nouvelles Archives*, 4, 1–50.

Bouvier, E. L. (1905). Monographie des Onychophores. *Annales Des Sciences Naturales. Zoologie Et Biologie Animale*, 2, 1–383.

Brito, S. V., Pereira, J. C., Ferreira, F. S., Vasconcellos, A. & Almeida, W. O. (2010). *Epiperipatus cratensis* sp. nov. (Onychophora: Peripatidae) from northeastern Brazil. *Neotropical Biology and Conservation*, 5, 47-52.

Brues, C. T. (1911). A new species of *Peripatus* from Grenada, with observations on other species of the genus. *Bulletin of the Museum of Comparative Zoology*, 55, 305–318.

Brues, C. T. (1914). A new *Peripatus* from Colombia. *Bulletin of the Museum of Comparative Zoology*, 58, 375–382.

Clark, A. H. (1913) Piccole note su degli Onychophora. *Zoologischer Anzeiger*, 42, 253–255.

Froehlich, C. G. (1962). A *Peripatus* from Barbados. *Boletim da Faculdade de Filosofia Ciências e Letras Universidade de São Paulo, Serie Zoologia*, 261, 325–334.

Froehlich, C. G. (1968). On some Brazilian Onychophores. *Beitrage zur Neotropischen Fauna*, 5, 160–171.

Marcus, E. & Marcus, E. (1955). A new *Peripatus* from Minas Gerais, Brazil. *Anais da Academia Brasileira de Ciências*, 27, 189–193.

Morera-Brenes, B. & Monge-Nájera, J. (1990). *Epiperipatus hilkae*, n. sp. from Costa Rica (Onychophora: Peripatidae). *Revista de Biología Tropical*, 38, 449–455.

Oliveira, I. S. & Wieloch, A. H. (2005). *Macroperipatus machadoi* sp. n. (Onychophora: Peripatidae) da Floresta Atlântica de Minas Gerais, Brasil. *Lundiana*, 6, 61–66.

Oliveira, I. S., Wieloch, A. H. & Mayer, G. (2010). Revised taxonomy and redescription of two species of the Peripatidae (Onychophora) from Brazil: a step towards consistent terminology of morphological characters. *Zootaxa*, 2492, 16–34.

Read, F. L. S. (1988a). The application of scanning electron microscopy to the systematics of the neotropical Peripatidae (Onychophora). *Zoological Journal of the Linnean Society*, 93, 187–223.

Read, F. L. S. (1988b). The Onychophora of Trinidad, Tobago and the Lesser Antilles. *Zoological Journal of the Linnean Society*, 93, 225–257.

Sclater, W. L. (1888). On the early stages of the development of a South American species of *Peripatus*. *Quarterly Journal of Microscopical Science*, 28, 343–363.

Scorza, J. V. (1953). Contribución al estudio de los Peripatus Caribes (Epiperipatus) de Venezuela, con adición de una nueva especie. *Revista de Sanidad y Asistencia Social*, 18, 783–788.

Sedgwick, A. (1888). A monograph on the species and distribution of the genus *Peripatus* (Guilding). *Quarterly Journal of Microscopical Science*, 28, 431–493.
