# Supplementary material for: Cryptic Speciation in Brazilian Epiperipatus (Onychophora: Peripatidae) Reveals an Underestimated Diversity among the Peripatid Velvet Worms
Source: PLoS One. 2011 Jun 10;6(6):e19973. doi: 10.1371/journal.pone.0019973 (PMC3112143; doi:10.1371/journal.pone.0019973)
Supplement: Table S2 — Summary of statistics for sequence data. (DOC) [file pone.0019973.s012.doc]

**Table S2.** Summary of statistics for sequence data.

| **Marker** | **Aligned sites** | **Base frequencies** | | | | | **Variable sites** | |
| --- | --- | --- | --- | --- | --- | --- | --- | --- |
|  |  | **A** | **C** | **G** | **T** | **A + T** | **Total** | **MP^**^** |
| *12S rRNA* | 340 | 0.36 | 0.09 | 0.13 | 0.42 | 0.78 | 93 | 61 |
| *COI* (1st^*^) | 194 | 0.26 | 0.13 | 0.25 | 0.35 | 0.51 | 32 | 25 |
| *COI* (2nd^*^) | 194 | 0.14 | 0.22 | 0.17 | 0.46 | 0.60 | 19 | 16 |
| *COI* (3rd^*^) | 194 | 0.40 | 0.03 | 0.06 | 0.51 | 0.91 | 108 | 70 |
| *COI* (total) | 582 | 0.27 | 0.13 | 0.16 | 0.44 | 0.71 | 159 | 111 |

^*^Codon position. ^**^Maximum Parsimony informative sites.
