# Supplementary material for: Cryptic Speciation in Brazilian Epiperipatus (Onychophora: Peripatidae) Reveals an Underestimated Diversity among the Peripatid Velvet Worms
Source: PLoS One. 2011 Jun 10;6(6):e19973. doi: 10.1371/journal.pone.0019973 (PMC3112143; doi:10.1371/journal.pone.0019973)
Supplement: Table S3 — Average genetic distances within and between onychophoran taxa ( COI + 12S rRNA ) according to the Kimura 2-parameter model. (DOC) [file pone.0019973.s013.doc]

**Table S3.** Average genetic distances within and between onychophoran taxa (*COI* + *12S rRNA*) according to the Kimura 2-parameter model.

|  | **Within taxa** | ***E. machadoi*** | ***E. diadenoproctus* sp. nov.** | ***E. paurognostus* sp. nov.** | ***E. adenocryptus* sp. nov.** |
| --- | --- | --- | --- | --- | --- |
| ***E. machadoi*** | 0.014 | - |  |  |  |
| ***E. diadenoproctus* sp. nov.** | 0.011 | 0.090 | - |  |  |
| ***E. paurognostus* sp. nov.** | 0.010 | 0.081 | 0.096 | - |  |
| ***E. adenocryptus* sp. nov.** | 0.020 | 0.091 | 0.072 | 0.044 | - |
| ***E. biolleyi*** | - | 0.174 | 0.185 | 0.183 | 0.186 |
